# Elements in Soft Tissues of the Young Mediterranean Mussel *Mytilus galloprovincialis* Lam. 1819 Collected in Sevastopol Bay (Crimea, Black Sea): Effects of Age, Sex, Location, and Principal Morphometric Parameters

**DOI:** 10.3390/ani13121950

**Published:** 2023-06-10

**Authors:** Sergey V. Kapranov, Alexander F. Kozintsev, Nikolay I. Bobko, Vitaliy I. Ryabushko

**Affiliations:** A.O. Kovalevsky Institute of Biology of the Southern Seas of RAS, 2 Nakhimov Ave., Sevastopol 299011, Russia

**Keywords:** bivalves, trace elements, ICP-MS, morphometry, multivariate analysis

## Abstract

**Simple Summary:**

Mussels accumulate trace elements in their soft tissues to levels several orders of magnitude higher than in the environment. For this reason, they are regarded as good trace element pollution bioindicators. Yet, there is limited understanding regarding which characteristics of mussels exhibit the strongest correlation with element accumulation. Furthermore, it remains unclear on what minimal spatial scale the differences in accumulation can be observed. We studied the effects of several biological characteristics on the contents of 72 elements in the soft tissues of mussels sampled from Sevastopol Bay. We found that most of the contents decreased with age, which was consistent with the decrease in the intercellular water content. There were significant differences in the element accumulation patterns in mussels from different sites of this relatively small water body, and multivariate statistical methods allowed distinguishing individuals sampled from each particular site. Sex differences did not significantly affect the overall element accumulation. Most elements demonstrated significant correlations as a function of only two gravimetric parameters, namely the ratio of soft tissue dry weight and the total weight of the mollusk. The results obtained contribute to understanding elemental biochemistry and aging in mussels and are of interest for improving mussel biomonitoring programs.

**Abstract:**

Although the mussel *Mytilus galloprovincialis* has been known for decades as an excellent bioindicator of trace element pollution in the marine environment, there is still no information on the effects of a suite of its principal morphometric parameters and age on trace element levels in soft tissues. In this work, using inductively coupled plasma mass spectrometry, we studied the contents of 72 elements in soft tissues of *M. galloprovincialis* aged 0.5–4, which were sampled at three stations within a relatively small water body, Sevastopol Bay. Significant effects of age and sampling location on the element contents and soft tissue dry-to-wet weight ratio were discovered. The effects of sex were not significant. It was presumed for the first time that the decrease in element content in the soft tissues of young mussels can be associated with the decrease in physiological needs for elevated contents of essential elements and intracellular water with age. Combinations of six principal morphometric parameters showed that a function of as few as three parameters (soft tissue dry weight, whole mollusk weight, and shell height, with by far the greatest contribution of the dry-to-total weight ratio) formed significant correlations with the contents of the largest possible number of elements (69–88% of the total number). For the first time, it was shown that linear discriminant analysis and canonical analysis of principal coordinates can be successfully used for tracing the exact origin of mussel samples within such a small water area. Canonical analysis of principal coordinates proved superior in the correct classification of the samples.

## 1. Introduction

Trace element pollution in seawater has been of great public concern for decades. In pristine oceanic waters, natural concentrations of most trace elements, including heavy metals, are extremely low [1]. However, great amounts of toxic trace element pollutants enter the marine environment as a result of anthropogenic activity. This poses risks to the health of marine biota and its consumers, including humans [2,3].

There are several methods for monitoring water pollution, with chemical and biological ones being the most widespread and popular [4]. Biological monitoring of marine ecosystem pollution is performed using indicator species, which are sensitive organisms that adequately respond to the anthropogenic impact in their habitat. At present, a large number of universal environmental pollution bioindicator species belonging to different taxonomic groups of biota are known [5,6]. Marine bivalves make up one of these groups [7].

The bivalve *Mytilus galloprovincialis* Lam. 1819 is indigenous to shelf zones of the Mediterranean and Black Seas [8,9]. At present, this mussel has spread throughout the temperate shelf waters of almost all oceans. It can exist in wide ranges of salinity (8–40‰) and temperature (1–28 °C). The optimum temperature is 12–20 °C and salinity is 12–25‰ [10,11]. This mussel has high growth rates; its shell can grow up to 140 mm in length, 75 mm in height, and 52 mm in width [12,13]. It lives 15–28 years but reaches terminal size much earlier [14]. Its long lifespan, sedentary lifestyle, relative ease of sampling, and ability to accumulate significant amounts of pollutants, in particular heavy metals, make this animal a convenient bioindicator for assessing the quality of coastal waters [15,16,17,18].

In a number of studies [19,20,21,22,23,24], the effect of the sampling site on trace element accumulation in mussels was investigated in the Mediterranean and Atlantic coastal areas. The trace element levels in soft tissues of *M. galloprovincialis* from different areas of the Black Sea also demonstrated significant spatial variability [25,26,27,28,29,30,31,32,33,34,35,36,37,38,39,40,41,42], especially for the animals of older age groups [43,44]. Typically, the authors compare pristine and polluted areas in terms of trace element abundance in soft tissues. However, it is also important to discern element abundance differences in groups of mussels sampled from different areas of a smaller and relatively polluted water body that is still large enough to differ in the trace element loads in its individual areas. An example of such a water body is the landlocked Sevastopol Bay in the Black Sea. In this study, three marginal bays within Sevastopol Bay were selected as sampling sites for mussels. The information on trace element pollution in Sevastopol Bay is ambiguous [45,46], which is apparently related to multiple pollution sources with varying contributions. As a result, there are highly variable spatiotemporal patterns of trace element concentrations. In this regard, environmental biomonitoring using the mussel *M. galloprovincialis* is one of the most appropriate biomonitoring strategies in such a water body over a medium-term scale. On the other hand, it is important to find out differences among the areas with comparable pollution level index (PLI) values, especially relatively low ones. On the southern coast of Sevastopol Bay, such areas are Martynova and Artillery bays and the estuary of the Chernaya River at Inkerman Bay, where PLI determined from the measurements of Mn, Cu, and Zn in sediments was about 2.0 [47].

The accumulation of trace elements in marine organisms is known to depend on a multitude of physicochemical and biological factors. The specific physicochemical factors affecting element bioaccumulation are temperature, water hardness or salinity, pH, organic carbon, dissolved oxygen, sediment grain size, and the hydrodynamic situation in the ecotope [48]. The biological factors within an individual species include age, sex, size, genotype, phenotype, feeding activity, and gonadal maturation stage [48,49,50,51,52]. The significance of morphometric parameters such as size and weight in trace element accumulation was recognized as early as at the time of the inception of the “Mussel Watch” biomonitoring programs in the 1970s [53,54]. At the same time, it was realized that size may be a less important factor for the element accumulation in tissues than sampling location (i.e., the degree of environmental pollution at different stations) and season [55]. However, regardless of the season and location, small mussels tended to contain higher levels of trace elements than big ones [54,55,56]. Therefore, small mussels can be more sensitive indicators of trace element pollution in seawater. Furthermore, as mussels in the Black Sea area are popular seafood and they reach marketable size (5–7 cm) within two years after attaching to the substrate [57], monitoring the element accumulation in mussels during this life period is of the utmost importance in terms of human health and safety.

In a previous study [58], the authors studied the effects of sex and some morphometric indices (condition index, soft tissue dry weight, and width-to-height ratio) on the abundance of 24 elements in the soft tissues of *M. edulis*. They found that the factor forming significant negative correlations with the abundance of the largest number of elements was the condition index. The authors attributed that to the fact that soft tissues tend to grow faster than elements are accumulated in them, thereby causing element dilution with biomass, and the condition index is the variable that is congruent with this process. However, no rigorous grounding was proposed as to why only the four variables in the selected algebraic forms had been chosen to form correlations with the element abundances. Another question not addressed in [58] or elsewhere, which is of considerable academic and applied importance in environmental biomonitoring, is: How many (and which) primary morphometric parameters in combination are sufficient to account for all possible correlations with the element contents in mussel soft tissues?

Given the above issues, the emphasis in this study is on trace element contents in soft tissues of young (0.5–4-year-old) individuals of *M. galloprovincialis* living in three different locations in Sevastopol Bay (Black Sea, Crimea) in relation to the individual age, sex, and six principal morphometric parameters (three allometric and three gravimetric ones) of the animals.

## 2. Materials and Methods

### 2.1. Sampling Area Characteristics

**Figure 1:**
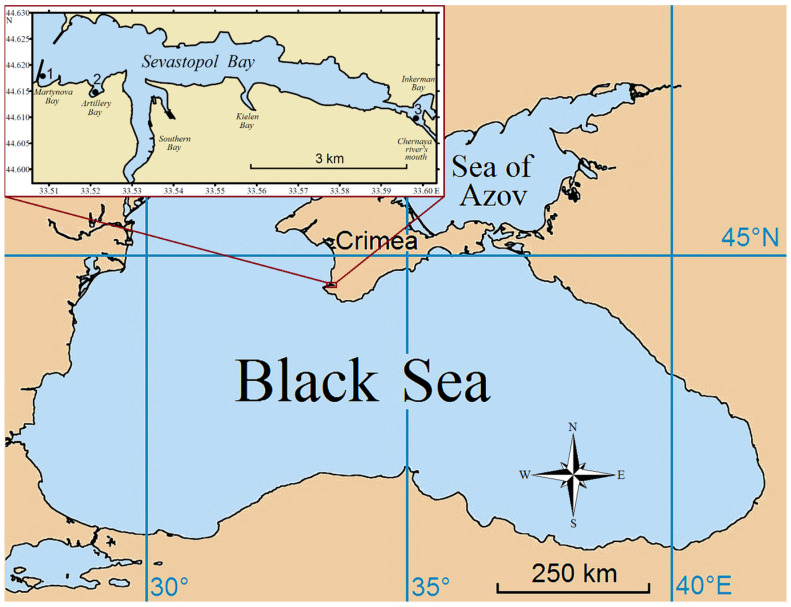
Map of the sampling stations 1–3 in Sevastopol Bay (inset).

The city of Sevastopol is located in the southwestern part of the Crimean Peninsula (Figure 1). Sevastopol Bay is a semi-enclosed water area with restricted water exchange that is about 7.5 km long and at most 1 km wide. It stretches from west to east and divides the city of Sevastopol into the northern and southern districts. The total area of the bay is 7.62 km^2^, and the total water volume in it is approximately 0.098 km^3^. The southern side of Sevastopol Bay is indented with several marginal ones: Martynova Bay, Artillery Bay, Southern Bay, and Kielen Bay. The depth at the bay mouth reaches 20 m and gradually decreases to 4–5 m in the innermost part [59]. The relative shallowness of the bay is responsible for the weakness of vertical stratification. Breezes and mountain valley winds, as well as nightly cooling, ensure a relatively strong vertical circulation and high thermohaline homogeneity of water. The large longitudinal extension of the bay leads to the formation of wind-generated currents and slopes of the water surface due to the tangential wind stress [60]. Variations in the surface slopes and the loss of water in the bay due to evaporation bring about compensatory inflows of Black Sea water from the outer roadstead and intensification of the water circulation through the mouth of the bay. The prevailing easterly winds (18% occurrence) contribute to the removal of water from the bay, while southerly and southeasterly winds (8–10%) intensify seawater inflow into the bay [59].

Sevastopol Bay is a water area of the estuarine type that is under permanent technogenic pressure (due to navigation, ship moorings, and hydraulic engineering works) and anthropogenic influence (through domestic urban discharge and pollutant-carrying rainfall-runoff). Pollution of this water area occurs due to the release of increasing amounts of insufficiently treated or untreated wastewater and emergency discharges [45,61]. The waters of the Chernaya River, which flow into the innermost part of Sevastopol Bay in the Inkerman area, are an additional and significant source of nutrients and other pollutants [62]. Although the influence of river runoff on the thermohaline structure of the water is undeniable, the main factor that determines the water circulation in the bay is wind. Due to this factor, a change in the spatial distribution of temperature and salinity occurs within several hours. The water exchange rate through the bay mouth is estimated at about 245 m^3^·s^−1^, with a 98 h period of full water replacement [63,64].

Depending on the localization of wastewater and rainfall outlets and on hydrometeorological conditions, both relatively unpolluted zones and zones with permanently high pollution levels are formed within Sevastopol Bay. In the study [59], the bay was divided into four areas. The least polluted part was the western part, and the moderately polluted part was the eastern part of the bay. The central part is the zone of heavy pollution, and Southern Bay is the most severely polluted area in Sevastopol Bay. The heavy metal concentrations in Sevastopol Bay vary widely from site to site. For Zn, this range was one order of magnitude larger, from 4.6 to 49.7 μg·L^−1^ [65]. At the same time, different metals tend to be more concentrated in different areas of the bay [66]. Overall, the seaward area of the bay was reported to be the least polluted with heavy metals, whereas the central part accumulated significant amounts. This is due to the existence of a quasi-stationary gyre in the central area [47,67], in which the pollutants are entrained. In addition, in the southeast corner of the bay, there is a shipbreaking plant and a city power plant, which contribute to the heavy metal contamination in this area. The heavy metal pollution of sediments in the bay was reported to increase from west to east [45]. However, a recent study [46] does not confirm this tendency, at least as regards such elements as Pb, Zn, and Cu.

### 2.2. Mussel Sampling and Morphometry

In this study, mussels were randomly sampled from three sites on the southern coast of Sevastopol Bay (Figure 1) in January 2020. Station 1 (44°37′4.34″ N 33°30′22.76″ E) was the inner side of the southern mole in Martynova Bay; Station 2 (44°36′49.60″ N 33°31′10.30″ E) was a boat mooring wall in Artillery Bay; and Station 3 (44°36′31.12″ N 33°35′48.21″ E) was a pier in the eastern part of Sevastopol Bay close to the Inkerman locality.

Mussels were collected manually with a scraper at a depth of 1 m. The collected mussels were packed in plastic bags and transported to the laboratory. From each sampling site, 40 mussel individuals belonging to 4–5 age groups (with approximately equal numbers of individuals in each) were selected for further research. In the laboratory, mussels were cleaned in seawater from epiphytes, epifauna, and mineral residues using a knife. The allometric parameters (shell length, height, and width) were measured using a caliper with an accuracy of 0.1 mm. Then, the total wet weight of the mollusk was measured on an analytical balance with a precision of 10^−4^ g. Mussel shells were opened with a plastic scalpel, and soft tissues were separated from the shells. Shell liquor was discarded. Gonadal smears were examined under a light microscope to determine sex and gonadal ripening stage. All mussels were at gonadal ripening stages 1, 3, 4, and 5, whose predominant occurrence is typical for this season in the northern Black Sea [68]. The soft tissues were blotted with filter paper and weighed with a precision of 10^−4^ g. The age of individual mussels was determined with an accuracy of half a year by counting light and dark bands in the shell cut [69]. Soft tissues were dried at 105 °C to a constant weight, which was registered with a precision of 10^−4^ g, and then homogenized in a porcelain mortar. All manipulations with sampled mussels were carried out within one day.

### 2.3. Analytical Sample Preparation

Samples of dried homogenized soft tissues (0.10 g) were put in PTFE tubes, into which 4 mL portions of the digestion medium were added. The digestion medium was 65% nitric acid of analytical grade, additionally purified in an acid purification system DST-1000 (Savillex, Eden Prairie, MN, USA). The capped tubes were let sit overnight and then autoclaved at a pressure of 2 bar for 1.5 h. The digested samples were diluted in polyethylene tubes with deionized water to 1000 mL·g^−1^ dry weight (d.w.).

### 2.4. Element Analysis

Concentrations of 72 elements were measured in the diluted samples using inductively coupled plasma mass spectrometry (ICP-MS) on a single-quadrupole instrument, PlasmaQuant^®^ MS Elite (Analytik Jena, Jena, Germany). The calibration curves were obtained using the multielement standards IV-ICPMS-71A–D (Inorganic Ventures, Christiansburg, VA, USA). The detection limits in this method range from 0.003 (^165^Ho) to 76 (^57^Fe) ng·L^−1^ [70].

The plasma flow was 9 L·min^−1^, the sampling depth was 7 mm, and the RF power was 1.25 kW. The dwell time for the trace elements was 10 ms, and for the macroelements (Na, Mg, Al, Si, K, and Ca) it was 1 ms. The scans were recorded in peak-hopping mode. Additional use of the collision reaction interface (CRI) in the ICP-MS analysis allows for eliminating polyatomic interferences and enhancing the linearity of calibration curves at the expense of signal strength (sensitivity). In the CRI mode “on”, gaseous hydrogen with a flow rate of 40 mL·min^−1^ was skimmer gas. No internal standard was used, as the matrix effects were expected to be insignificant. The signal drift was compensated by measuring the apparent element concentrations in the standard solution IV-ICPMS-71A (20 μg·L^−1^) after every tenth sample and using an interpolating polynomial correction function based on the values obtained [36]. Fluorine concentration was calculated semi-quantitatively using the mass-19 signal and calibrated signals of analytes. The quantitation error in semi-quantitative ICP-MS analysis is typically less than 50% [71,72].

The accuracy of the analysis was verified by measuring the element concentrations in the certified European Reference Material ERM^®^-CE278k (tissue of the mussel *Mytilus edulis* Linnaeus, 1758). Samples of the reference material (0.1 g) were digested in extra-pure nitric acid and diluted with deionized water according to the procedure described above. The certified and registered values are given in Supplementary Table S1.

### 2.5. Statistical Analyses and Correlations

Effects of age, sex, and sampling location on element contents and morphometric indices were tested using three-way permutational multivariate analysis of variance (PERMANOVA) in PRIMER 6.1.16 and PERMANOVA 1.0.6 [73,74]. The same software was used to perform principal component analysis and canonical analysis of principal coordinates. Linear discriminant analysis [75,76] and the Shapiro–Wilk test for normality were performed in PAST 4.11 [77]. The significance level in all statistical procedures was adopted at p<0.05. Some basic concepts and relationships for the multivariate analyses used in this work are presented in the Supplementary Material.

Boxplots were produced in Matlab 8.2.0 using the boxplot function. They include: a red horizontal line of the median ($q_{2}$, second quantile); blue horizontal lines of the 25th ($q_{1}$, first quantile) and 75th ($q_{3}$, third quantile) percentiles; whiskers of the expected minimum and maximum calculated as $q_{1}-1.5\;\left(q_{3}-q_{1}\right)$ and $q_{1}+1.5\;\left(q_{3}-q_{1}\right)$, respectively, stretching to the farthest values within the range between the expected minimum and maximum (min–max); triangular notches roughly corresponding to the 95% confidence interval of the median, whose limits are calculated as $q_{2}-1.57\;\left(q_{3}-q_{1}\right)/\sqrt{n}$ and $q_{2}+1.57\;\left(q_{3}-q_{1}\right)/\sqrt{n}$; and outliers as red crosses that lie beyond the min–max range.

To find the principal morphometric parameters correlating with the contents of as many elements as possible, the following functions were constructed as the products of all combinations of the morphometric parameters raised to corresponding powers:

(1) $$F_{n}=\prod_{i=1}^{n=1,2,\ldots ,6}P_{i}^{x_{i}}$$

where $P_{i}$ denotes any of the six parameters: shell length *L*, width *D*, height *H* [78], soft tissue wet weight *WW*, dry weight *DW*, and whole mollusk weight *W*.

Pearson’s and Spearman’s correlations of the functions $F_{n}$ (1) and the element contents were calculated in Matlab 8.2.0 (MathWorks, Natick, MA, USA) using the *corr* function with the corresponding arguments. The exponents in Equation (1) that resulted in significant correlations (p<0.05) were sampled randomly using the *rand* function from the range (−10, 10). After 1000 exponent combinations had been found, only those giving correlations with the largest number of elements (*N*) were selected. Additionally, the exponent sampling was repeated until *N* stopped growing and the number of exponent combinations found for this *N* reached 10,000.

As the exponents for Pearson’s correlations with the largest number of element contents are found within limited ranges, the median was taken as the most probable value (except for n=1). The exponents for all Spearman’s correlations and Pearson’s correlation at n=1 were found using a bootstrap procedure, as no distinct maximum can be spotted in their distributions. This procedure involves randomly selecting fixed-size samples, from which exponent values with the largest occurrences are extracted and accumulated in an array. The median in this array was considered the most probable exponent. In the case of more than one set of elements found for the same *N*, only those were picked out from Pearson’s and Spearman’s correlations that gave the closest exponent values in both.

## 3. Results and Discussion

### 3.1. Element Contents

The element quantitation results for mussels from three sampling sites are summarized in Table 1 as the median, minimum, and maximum values. The pooled data for each station demonstrated heavy-tailed distributions that were significantly non-normal for most elements under consideration, as follows from the Shapiro–Wilk test results. The most abundant element was sodium (2.8–13.3 g·kg^−1^ d.w.), and the least abundant one was rhenium (below 0.9 μg·kg^−1^ d.w. in any sample).

The comparison of our results with those of other authors on the element contents in *M. galloprovincialis* from the Black Sea (Table 2) shows that many elements (Cr, Mn, Fe, Cu, Se, Cd, Hg, and Pb) in our research demonstrate levels that are >20% higher than the regional medians. This indicates the overall pollution of Sevastopol Bay with trace elements, among which there are notoriously toxic heavy metals: Cd, Hg, and Pb. Only one metal (V) was at a level >20% lower than the medians among the elements whose contents could be compared (reported in at least five studies). The contents of a number of elements (K, Al, Co, Ni, Zn, As, and Sn) were approximately at median levels.

### 3.2. Effects of Sex, Location, and Age

A three-way global PERMANOVA applied to the standardized element contents in mussel samples shows that the effects of station location and mussel age are significant (*p* < 0.001), whereas the significance of the sex effect is marginal (*p* = 0.063, Supplementary Table S2). In the element-wise PERMANOVA, the contents of most of the elements under consideration (54 out of 72) revealed significant effects of sampling location, and 26 elements showed significant differences with age. At the same time, only seven elements (P, As, Y, Ag, Cd, In, and Sb) demonstrated sex-related differences. Except for P, which is more accumulated in male gonads [52] in the form of phosphate esters for powering sperm motility, these elements do not have any known essential function in the mussel organism. The other six elements were more accumulated in females, most likely due to the occasional incorporation of trace element-containing particles or diffusion from seawater in female gonads after spawning. In *Mytilus* females, the empty gonad compartments that are free of gametes and filled with seawater tend to be larger [81,82]. Consequently, there is a greater probability of capturing microparticles in female gonads. This presumption may account for the significant differences in levels of some elements between the sexes. The statistically significant enrichment of female gonads with a number of trace elements was also observed in the work [52].

**Figure 2:**
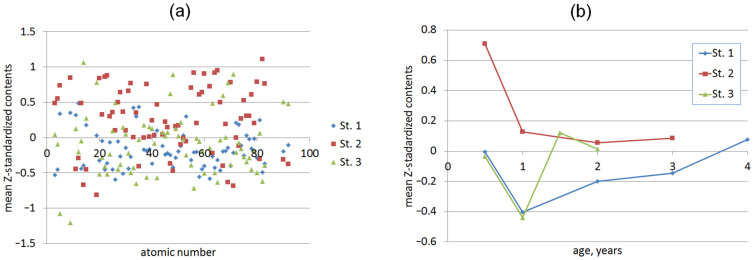
Mean Z-standardized element contents in soft tissues of mussels from the three stations vs. (**a**) atomic number of elements in the periodic table (the contents are age-averaged) and (**b**) age (the contents are element-averaged).

When analyzing Z-standardized element contents in mussels from the three stations (Figure 2), it can be noted that the mollusks from Station 2 were enriched with most of the elements. This enrichment can be attributed to the entry of these elements into Artillery Bay due to factors such as emergency wastewater discharge, rainfall sewer discharge, and intense marine traffic in this area [45,62]. Animals from Station 3 were more enriched with Si, P, K, Fe, Ag, Cd, Eu, Ho, Tm, Lu, Th, and U, which may originate from both the Chernaya river discharge (Si, P, K) and pollution due to specific human activities (Fe and Cd). The elements that are more abundant in mussels from the more seaward Station 1 are mainly the seawater solutes (Na, Mg, As, Br, I).

Among the age-related effects, in most cases, there was a sharp drop in the element contents after 0.5 years of age (Figure 2b and Supplementary Table S3). This is in agreement with the other authors’ observations of higher trace element levels in the soft tissues of smaller and younger mussels [38,54,83,84]. This phenomenon was attributed to the higher metabolic activity in young animals, resulting in a greater pumping of water volumes relative to their body weight. Additionally, their higher surface-to-volume ratio enables the absorption of greater amounts of dissolved elements per unit weight [54,84,85,86].

From the 1-year-old age, the abundance of many elements in mussels from Station 2 continued decreasing or remained nearly at the same level. Although at Stations 1 and 3, such patterns were observed in some cases, there were a much larger number of elements that were being steadily accumulated after the first year of mussel life. However, quite a few elements (Si, K, Lu, Os, and U) demonstrated significant and consistent age patterns at all three stations. This fact underlines that age *per se* is, at least, not a decisive factor in the element accumulation in mussels, in line with the results of the work [58].

### 3.3. Effects of Morphometric Parameters

As the decrease in element content is commonly attributed to biomass dilution, it is important to first follow variations of the gravimetric parameters with age.

**Figure 3:**
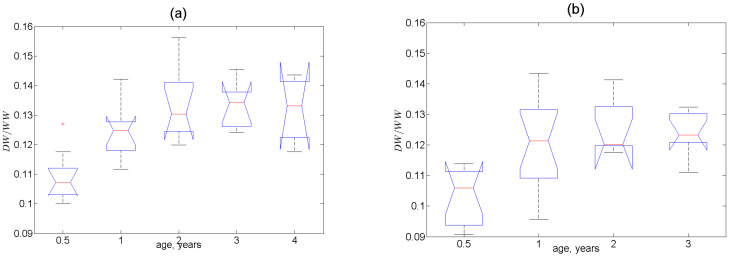

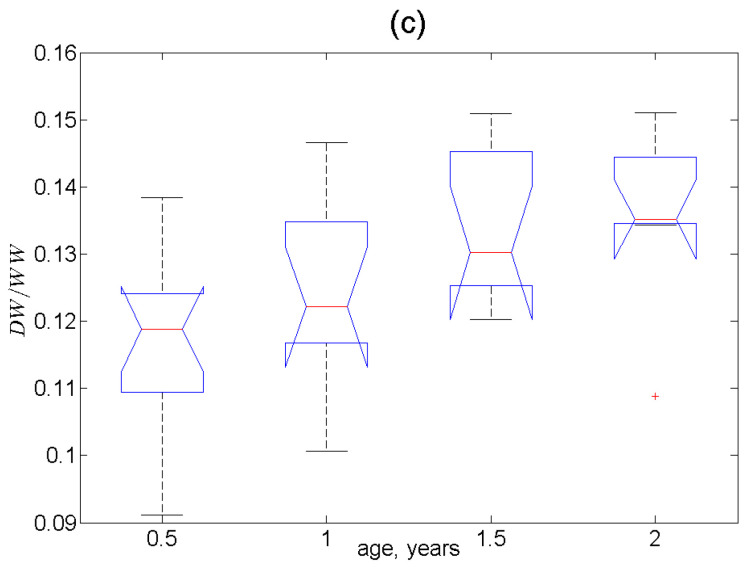
Boxplots of the ratios of dry (*DW*) and wet weight (*WW*) of soft tissues as a function of mussel age: (**a**) Station 1, (**b**) Station 2, (**c**) Station 3. Red crosses denote outliers.

The most indicative parameter that varied consistently with age at all three stations was the ratio of dry and wet weights of soft tissues. In Figure 3, it is seen that this ratio significantly increased until the 1–2-year-old age and then remained constant. The significance of the age (*p* < 0.001) and location (*p* = 0.005) effects and the lack of significance in the sex effects (*p* = 0.39) on DW/WW were also evidenced in the PERMANOVA test results (Supplementary Table S4). It is very noteworthy that the contents of most of the elements in Station-2 mussels and many elements in mussels from Stations 1 and 3 followed the age pattern of DW/WW mirrored with respect to the horizontal axis. Therefore, one should expect significant positive correlations between these contents and the values of 1−DW/WW. Indeed, for the pooled samples, there were 38 significant (*p* < 0.05) Pearson correlations, among which only five were negative (for Na, Si, P, K, and Lu—Supplementary Table S5). Additionally, there were 46 significant Spearman correlations, among which six were negative (for the macroelements Na, Mg, Si, P, K, and Br). The use of Spearman’s rank-order correlations should be considered more appropriate because most of the element dispersions are significantly non-normal.

In fact, function 1−DW/WW is the relative water content in soft tissues. It is worthwhile to note that the content of calcium, unlike other macroelements, positively correlates with 1−DW/WW, which means that the soft tissues of the youngest mussels contain more calcium per unit weight. Calcium is needed primarily for shell growth, and it is positively correlated with the greater content of intracellular water that is probably needed to compensate for increased osmotic pressure. One can assume that most of the elements giving positive correlations with this function are accumulated in the aqueous phase of soft tissues according to a similar mechanism. It should be noted that this mechanism differs from passive diffusion from seawater or seawater filling lacunae in the tissues because the other macroelements demonstrate a negative correlation with the relative water content. The increased content of water and many elements in the youngest mussels may be associated with their higher filtration and metabolism rates per unit weight [87,88]. This presupposes higher concentrations of essential elements needed to maintain higher metabolic levels.

Furthermore, it is of interest to find the minimum number of principal morphometric parameters needed to form a correlation with the largest number of element abundances in soft tissues. The largest number (*N*) of element abundances significantly correlating with the functions $F_{n}=\prod_{i=1}^{n=1,2,\ldots ,6}P_{i}^{x_{i}}$ in Equation (1) was found to increase with *n* (Table 3) up to n=3. The results of seeking the related exponents are shown in Supplementary Figures S1–S3. At n=3, the maximum number of elements (*N*_max_) was reached: 50 for Pearson’s and 63 for Spearman’s correlations. In both cases, the parameters involved were *H*, *DW*, and *W*. It is seen that the parameters in the numerators and denominators in Pearson’s *F*_3_ are inverse to those in Spearman’s *F*_3_. For this reason, the significant Pearson’s and Spearman’s correlation coefficients for any element in Table 1 have opposite signs.

Thus, 69–88% of the elements under study have significant correlations with as few as three morphometric parameters, two of them being gravimetric ones. At 3≤n≤6, *N* remains *N*_max_, and any combinations of parameters in *F_n_* yielding *N*_max_ significant correlations always include the above three parameters.

Interestingly, at n=2, *N* already reaches, at least, 0.9 *N*_max_, and most of the correlations with ${\left(DW/W\right)}^{0.7}$ are negative (Table 1 and Supplementary Table S5). From the form of this ratio, this could have been traditionally attributed to biomass dilution. However, taking into account the above-mentioned numerous correlations with the relative water content, one should rather expect that the lower whole mollusk weight, including the lower weight of intracellular water, at the same weight of dry soft tissues is the main reason for the decreasing contents of many elements.

In the case of one-parameter correlations (n=1), the largest number of elements correlate with $D^{-1.7}$. It is reasonable to assume that the one-parameter function $D^{-1.7}$ giving correlation coefficients with signs opposite to those of ${\left(DW/W\right)}^{0.7}$ (Supplementary Table S5) is the best approximation of ${\left(DW/W\right)}^{-0.7}$, i.e., the ratio DW/W varies with age in approximately the same manner as $D^{2.4}$.

The function *F*_3_, in addition to the weights ratio factor, contains also the ratio of the total weight and shell height raised to a power of 3.6–4.3. This factor may imitate, to some extent, the shell width-to-height ratio raised to a certain power. This ratio was shown [58] to be a significant factor in the correlation with the abundance of many elements in the soft tissues of *M. edulis* and was attributed to decreased growth rate and increased relative physiological age. This is in agreement with our observations of the sharp drop in most element abundances after 0.5 years of age.

Several elements do not demonstrate significant correlations with the function *F*_3_. Many of them (Fe, Cu, and Zn) are essential to mussel physiology. It is logical to assume that these elements are accumulated in physiologically necessary concentrations irrespective of age, intracellular water content, or biomass growth. This group also includes arsenic, a relatively abundant trace element, and it is an argument in favor of the existence of some essential functions of As in mussels. Previously, the possible essentiality of As was assumed in mussel oogenesis [52]. Other elements in this group are ultratrace elements (Ge, Ta, Tl) that could have been occasionally captured from the environment and thus have no relation to the morphometric parameters of the mussel body.

### 3.4. Multivariate Analysis

**Figure 4:**
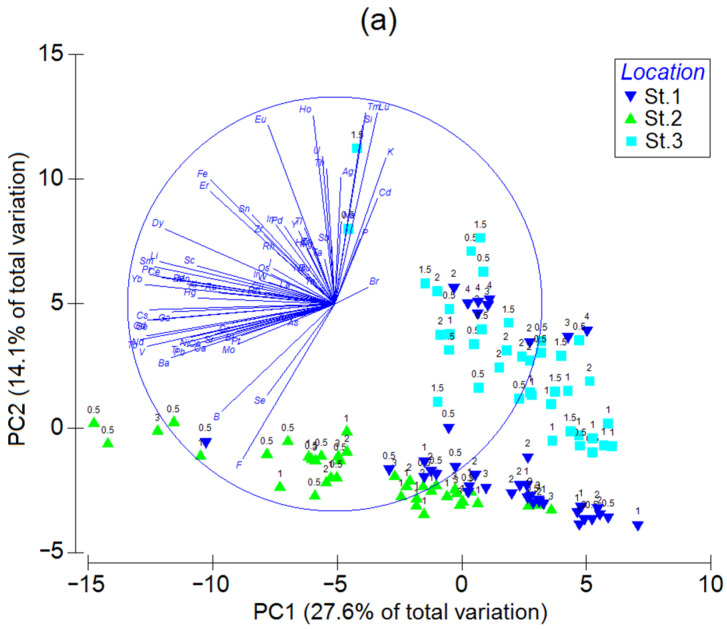

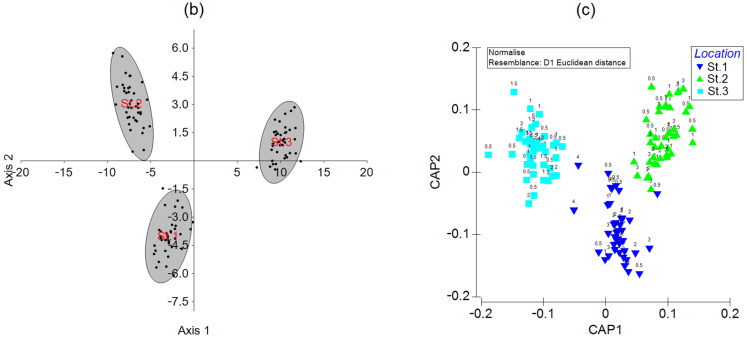
(**a**) Principal component analysis, (**b**) linear discriminant analysis, and (**c**) canonical analysis of principal coordinates of Z-standardized element contents in soft tissues of mussels from three stations in Sevastopol Bay. The numbers above the symbols in (**a**,**c**) denote individual mussel ages. Gray ellipses in (**b**) are 95% confidence areas for the groups.

The results of the principal component analysis with the Z-standardized element contents as variables and individual mussel samples as observations (Figure 4a) demonstrate that the first two principal components (PC1 and PC2) explain 27.6 and 14.1% of the total variation of the element abundances, respectively. The scores of the data from Stations 1 and 2 (with the smallest and greatest trace element abundance, respectively) are stretched along PC1. Thus, PC1 is associated mainly with the overall trace element contamination degree, with its positive half-space corresponding to the lower levels. The Station-3 data, together with some observations of Station-1 mussels aged 3 and 4, are scattered mostly in the PC2 direction. Apparently, this principal component is primarily related to the element’s accumulation with age. In the positive direction of PC2, there are vectors of Na, Si, K, P, Ag, Cd, and some lanthanides, which tend to be more accumulated in older mussels. Interestingly, the scores in the groups stretched along PC1 and PC2 can be fitted with two straight lines that intersect at PC1 ≈ 8. The intersection is the point that most likely corresponds to the lowest possible element levels in the soft tissues of mussels from Sevastopol Bay, irrespective of the exact sampling location.

Linear discriminant analysis (LDA) was performed to check whether the observations could be correctly assigned to the stations where the corresponding samples had been obtained. The LDA results are shown in Figure 4b as a projection onto a plane of the first two canonical axes. The data in the 95% confidence ellipses demonstrate the full separation of the groups in the canonical space. The maximum separation is observed between the data from Station 3 and Stations 1 and 2 along Axis 1, explaining 86% of the variation. The second largest separation is between Station 1 and Stations 2 and 3 along Axis 2, explaining 14% of the variation. The estimated and given group assignments coincided in 100% of the cases. The jackknifing (leave-one-out) cross-validation procedure gives 87.5% correctly classified observations (85% for Stations 1 and 2 and 92.5% for Station 3). Thus, LDA allows determining, with an accuracy of no lower than 85%, mussel sampling locations based on the element abundances in soft tissues.

Another promising technique for sample assignment to a certain location is a canonical analysis of principal coordinates (CAP) [74,89,90]. It also demonstrates a tight grouping of observations for each station (Figure 4c). The cross-validation reveals the total correct classification of 96.7% of the samples (95% for Station 1 and 97.5% for Stations 2 and 3), which implies that CAP is more powerful than LDA in terms of the correct assignment of observations to the geographic origin of mussels.

Discriminant analysis has already been successfully used for tracing the geographic origin of mytilids [91,92,93,94] and other bivalves [95,96]. However, in those works, element contents were analyzed almost exclusively in mollusk shells, and distances between sampling stations were much greater than those in the present study. The percentages of correct assignments were nearly the same as in our work. CAP applied to the element abundances in the shells of some clams [97,98] showed high accuracy in the correct classification of the clam shell samples. Principal component analysis was also applied to assign mussel samples to sampling locations [23,37,50,93,99,100,101], but with less success as this method is not perfectly intended for this purpose.

## 4. Conclusions

This work presents the first comparative study of the contents of 72 elements in the soft tissues of the mussel *M. galloprovincialis* collected in three zones of a relatively small (7.5 km long) and narrow water body (Sevastopol Bay). As compared with mussels from other areas of the Black Sea, mussels from Sevastopol Bay contain higher levels of many elements, including toxic heavy metals. This indicates the relatively strong trace element pollution in the bay. The contents of many elements indicate that the most polluted area among those under study is Artillery Bay (Station 2).

The element contents and a gravimetric index (soft tissue dry-to-wet weight ratio, DW/WW) have been considered in relation to mussel sex, age, and sampling location. The effects of age and sampling location on these parameters have been found to be significant, whereas the effects of sex have been shown to lack significance. Whatever the sampling location, the contents of most elements significantly decrease from 0.5- to 1-year-old age. An inverse pattern has been observed for DW/WW, with significant positive correlations between 1−DW/WW and the contents of many elements. These findings suggest that the increased intracellular water content, rather than the commonly proposed lower biomass dilution, is likely associated with the higher element levels in the soft tissues of younger mussels. This feature may be related to the increased filtration and metabolism rates and higher needs for essential elements, especially calcium, in young individuals.

For the first time, it has been shown that as few as three principal morphometric parameters (whole mollusk weight, dry weight of soft tissues, and shell height) in the form of products of their power-law functions are sufficient to form significant correlations with abundances of the largest possible number of elements. A maximum of 50 and 63 elements (i.e., 69.4 and 87.5% of the total number) have been found to yield significant Pearson’s and Spearman’s correlations with the functions of the morphometric parameters. At the same time, more than 90% of the significant correlations have been obtained with the combination of only two gravimetric parameters (dry-to-total weight ratio raised to the power of 0.7).

Principal component analysis (PCA) has demonstrated that the main factor determining differences in the element contents in soft tissues is ambient levels of elements in the surrounding environment. The second most important factor is related mainly to age. In PCA, groups of observations from different stations are not well separated, and this analysis is not very helpful in identifying sampling locations on such a short spatial scale. Yet, two other multivariate techniques—linear discriminant analysis (LDA) and canonical analysis of principal coordinates (CAP)—have proven to be very efficient in assigning mussel samples to particular sampling locations. It has been found that LDA allows correctly classifying at least 85% of samples, and the correct classification using CAP occurs in no fewer than 95% of cases.

The present work contributes to the understanding of the still obscure mechanisms and determinants of trace element accumulation in the mussel body. On the one hand, the limited number of morphological parameters affecting element accumulation, the insignificant sex effects, and the dubious effects of age as ordinary physicochemical accumulation in time (without regard to concomitant morphological and biochemical transformations in the organism) facilitate these future studies within this field. On the other hand, the current understanding lacks clarity regarding the proportions in which the element abundances in young mussels decrease as a result of biomass dilution and the physiological decrease in the need for elevated concentrations of essential elements and intracellular water with age. This issue requires further research.

## Figures and Tables

**Table 1:** Median (min–max) values of the element contents (in mg·kg^−1^ d.w.) in mussels from three stations in Sevastopol Bay; significance (+) of sex, sampling location, and age effects in relation to the element contents; and coefficients of Pearson’s and Spearman’s correlations with *F*_2_ and *F*_3_ (Equation (1)).

				Effects			Correlation with *F*_2_		Correlation with *F*_3_	
	Station 1	Station 2	Station 3	Sex	Loc	Age	Pearson	Spearman	Pearson	Spearman
Li	1.38 (0.73–2.88)	1.92 (1.13–4.49)	1.76 (0.95–3.35)	-	+	-	−0.32	−0.29	0.34	−0.20
Be	0.015 (0.006–0.032)	0.022 (0.012–0.046)	0.015 (0.009–0.063)	-	+	-	−0.29	−0.34	0.25	−0.30
B	13.2 (7.4–18.8)	13.7 (11.5–17.6)	8.2 (3.1–13.4)	-	+	+	−0.35	−0.37	0.29	−0.41
F *	134 (6–191)	144 (120–179)	25 (BDL—40)	-	+	+	−0.47	−0.47	0.44	−0.54
Na	7199 (5492–11,209)	6171 (4761–10,605)	7230 (2820–13,295)	-	+	+	0.21	0.25	−0.26	0.27
Mg	640 (478–972)	558 (427–650)	585 (230–993)	-	+	+	0.19	0.29	−0.25	0.28
Al	131 (45–346)	263 (110–781)	162 (74–1286)	-	+	-	−0.18	−0.25	0.28	−0.22
Si	27 (14–118)	26 (16–76)	106 (20–194)	-	+	+	0.37	0.39	−0.37	0.46
P	6291 (3801–11,198)	5038 (3862–7636)	6655 (2101–13,095)	+	+	+	0.47	0.43	−0.37	0.39
K	6014 (4052–8328)	4718 (3798–5973)	7837 (2598–12,060)	-	+	+	0.54	0.59	−0.48	0.62
Ca	338 (137–997)	722 (274–2430)	275 (142–580)	-	+	-	−0.35	−0.50	0.38	−0.54
Sc	0.27 (0.13–0.41)	0.29 (0.20–0.43)	0.27 (0.15–0.36)	-	+	-	−0.20	−0.18	0.19	−0.19
Ti	2.1 (1.1–4.9)	3.8 (1.8–14.1)	2.2 (1.4–4.4)	-	+	-	−0.38	−0.48	0.38	−0.45
V	0.69 (0.24–1.89)	1.33 (0.35–3.53)	0.60 (0.18–1.35)	-	+	+	−0.45	−0.46	0.49	−0.46
Cr	2.0 (0.4–8.5)	2.4 (1.3–5.0)	1.8 (0.7–5.8)	-	-	-	−0.31	−0.33	0.28	−0.26
Mn	11.8 (5.6–23.0)	15.5 (7.8–33.5)	13.3 (6.7–38.1)	-	+	-			0.18	
Fe	247 (115–495)	321 (159–928)	385 (162–796)	-	+	-				
Co	0.71 (0.42–2.02)	0.90 (0.59–1.29)	0.67 (0.23–1.07)	-	+	-	−0.37	−0.42	0.35	−0.38
Ni	2.3 (1.0–6.0)	3.6 (1.5–6.6)	2.2 (BDL—8.4)	-	+	+	−0.36	−0.43	0.40	−0.38
Cu	107 (17–212)	181 (77–311)	149 (26–744)	-	+	-		−0.24		
Zn	94 (42–387)	128 (39–271)	125 (46–248)	-	-	-		−0.34		
Ga	0.09 (0.03–0.19)	0.17 (0.05–0.47)	0.11 (0.02–0.46)	-	+	-	−0.28	−0.43	0.37	−0.32
Ge	0.15 (0.11–0.28)	0.19 (0.13–0.43)	0.14 (0.08–0.20)	-	+	-	−0.40		0.38	−0.41
As	13.5 (7.5–21.5)	12.1 (7.5–18.7)	10.1 (5.2–16.3)	+	-	-				
Se	5.6 (1.7–8.7)	5.5 (4.3–6.8)	3.8 (2.0–7.1)	-	+	+	−0.27	−0.28	0.22	−0.27
Br	120 (75–186)	75 (50–133)	88 (22–412)	-	+	-		0.18	−0.18	0.18
Rb	4.4 (2.7–6.3)	4.4 (3.3–6.8)	4.9 (2.1–7.9)	-	-	+				0.19
Sr	14 (11–26)	19 (12–49)	12 (7–19)	-	+	-	−0.33	−0.43	0.35	−0.47
Y	0.16 (0.04–0.33)	0.22 (0.12–0.42)	0.11 (0.01–3.78)	+	-	+		−0.35		−0.40
Zr	0.21 (0.05–1.16)	0.43 (0.08–1.05)	0.29 (0.05–1.95)	-	+	+		−0.19		−0.21
Nb	0.005 (0.001–0.437)	0.014 (0.003–0.279)	0.008 (0.001–0.141)	-	-	-			0.22	−0.23
Mo	0.94 (0.42–1.64)	0.97 (0.64–1.84)	0.63 (0.13–2.38)	-	+	+	−0.33	−0.41	0.33	−0.41
Ru	4 × 10^−4^ (BDL—0.022)	0.001 (BDL—0.037)	0.002 (BDL—0.019)	-	-	-		0.23		0.26
Rh	0.014 (0.003–0.026)	0.024 (0.012–0.041)	0.011 (0.002–0.245)	-	-	-		−0.46		−0.43
Pd	0.03 (0.0005–0.43)	0.07 (0.03–0.13)	0.03 (0.00–0.73)	-	-	-		−0.37		−0.39
Ag	0.46 (0.20–16.87)	0.54 (0.25–6.89)	2.69 (0.38–20.9)	+	+	+				0.18
Cd	2.1 (1.3–6.8)	2.4 (1.8–3.1)	4.8 (0.7–10.8)	+	+	+	0.19		−0.19	0.26
In	6 × 10^−4^ (BDL–0.0033)	0.0012 (6 × 10^−4^–0.0087)	9·10^−4^ (3 × 10^−4^–0.009)	+	-	+		−0.27		−0.25
Sn	0.07 (BDL—0.95)	0.21 (0.07–0.72)	0.07 (0.01–2.12)	-	-	-		−0.37		−0.39
Sb	0.03 (0.02–0.13)	0.05 (0.02–0.45)	0.03 (0.004–22.3)	+	-	+		−0.37		−0.35
Te	0.01 (0.006–0.45)	0.01 (0.007–0.17)	0.01 (0.0004–0.19)	-	-	-		−0.19	0.23	
I	18 (12–63)	17 (10–30)	11 (2–94)	-	-	-	−0.21	−0.33	0.19	−0.34
Cs	0.047 (0.016–0.099)	0.079 (0.018–0.192)	0.044 (0.009–0.121)	-	+	-	−0.38	−0.41	0.45	−0.41
Ba	2.3 (1.0–8.1)	4.4 (2.4–7.4)	1.8 (0.9–3.8)	-	+	-	−0.37	−0.47	0.36	−0.52
La	0.39 (0.03–2.80)	0.75 (0.31–1.40)	0.45 (0.13–4.49)	-	-	-	−0.19	−0.33		−0.28
Ce	0.29 (0.09–0.75)	0.51 (0.23–1.78)	0.38 (0.18–0.78)	-	+	-	−0.32	−0.35	0.36	−0.31
Pr	0.04 (0.014–0.09)	0.07 (0.03–0.20)	0.05 (0.02–0.11)	-	+	-	−0.35	−0.36	0.39	−0.34
Nd	0.19 (0.04–0.65)	0.40 (0.19–1.20)	0.19 (0.09–0.39)	-	+	-	−0.46	−0.54	0.48	−0.54
Sm	0.03 (BDL—0.11)	0.07 (0.03–0.18)	0.05 (0.02–0.11)	-	+	-	−0.36	−0.40	0.33	−0.37
Eu	0.010 (0.001–0.035)	0.012 (0.006–0.026)	0.018 (0.004–0.040)	-	+	+				
Gd	0.06 (0.013–0.14)	0.09 (0.05–0.20)	0.05 (0.03–0.10)	-	+	-	−0.42	−0.50	0.40	−0.50
Tb	0.005 (0.0005–0.012)	0.008 (0.004–0.018)	0.004 (0.002–0.008)	-	+	-	−0.48	−0.53	0.50	−0.54
Dy	0.03 (0.003–0.07)	0.05 (0.02–0.09)	0.04 (0.02–0.08)	-	+	+	−0.29	−0.32	0.24	−0.26
Ho	0.006 (BDL—0.031)	0.007 (0.004–0.015)	0.015 (0.005–0.033)	-	+	+	0.18		−0.20	0.20
Er	0.017 (0.003–0.054)	0.022 (0.012–0.044)	0.023 (BDL–0.053)	-	+	+	−0.18	−0.22		−0.19
Tm	0.0021 (BDL—0.026)	0.0027 (0.0013–0.0054)	0.013 (0.006–0.033)	-	+	+	0.31	0.30	−0.31	0.35
Yb	0.015 (0.003–0.038)	0.024 (0.012–0.046)	0.016 (0.007–0.029)	-	+	-	−0.38	−0.41	0.35	−0.39
Lu	0.0018 (BDL—0.028)	0.0026 (0.0013–0.0056)	0.0162 (0.0061–0.0370)	-	+	+	0.35	0.29	−0.35	0.35
Hf	0.008 (0.000–0.74)	0.023 (0.007–0.321)	0.017 (0.008–0.129)	-	-	-		−0.21		−0.22
Ta	0.001 (BDL—0.46)	0.003 (0.001–0.146)	0.003 (0.001–0.353)	-	-	-				
W	0.03 (BDL–0.41)	0.07 (0.03–0.40)	0.04 (0.01–0.46)	-	+	-	−0.33	−0.49	0.34	−0.49
Re	0.00007 (BDL—0.0009)	0.0002 (0.0001–0.0007)	0.0001 (BDL—0.0007)	-	+	-	−0.32	−0.50	0.26	−0.48
Os	0.001 (BDL—0.039)	0.003 (0.0011–0.078)	0.0004 (BDL—0.005)	-	+	-		−0.51	0.20	−0.56
Ir	0.0002 (BDL—0.0040)	0.0003 (0.0001–0.0046)	0.0001 (BDL—0.0034)	-	+	-		−0.41	0.18	−0.44
Pt	0.002 (BDL—0.029)	0.004 (0.002–0.018)	0.001 (BDL—0.004)	-	+	-	−0.42	−0.62	0.36	−0.64
Au	0.01 (BDL—5.38)	0.03 (0.013–15.4)	0.009 (0.004–0.09)	-	-	-		−0.52	0.36	−0.56
Hg	0.13 (0.07–0.37)	0.23 (0.08–0.35)	0.13 (0.08–0.32)	-	+	+	−0.40	−0.44	0.40	−0.45
Tl	0.021 (0.0011–0.043)	0.019 (BDL—0.035)	0.020 (0.010–0.038)	-	+	-				
Pb	5.7 (2.8–19.0)	12.6 (8.3–22.4)	5.4 (3.1–8.1)	-	+	-	−0.40	−0.40	0.34	−0.46
Bi	0.02 (BDL—0.06)	0.05 (0.015–0.34)	0.02 (0.01–0.04)	-	+	-	−0.37	−0.56	0.32	−0.59
Th	0.11 (0.03–0.76)	0.13 (0.07–0.66)	0.23 (0.14–0.92)	-	+	-		0.19		0.25
U	0.11 (0.07–0.86)	0.11 (0.07–0.47)	0.23 (0.12–0.68)	-	+	+				0.20

* measured semi-quantitatively.

**Table 2:** Median or mean element contents (mg·kg^−1^ d.w.) in soft tissues of *M. galloprovincialis* from different areas of the Black Sea coast (N = north, W = west, etc.) and from the northern coast of the Tyrrhenian Sea (Tyr) according to different studies. Italic emphasis (blue shadowing): values >20% lower than the median, bold emphasis (red shadowing): values >20% higher than the median.

	N	N	N	N	W	W	W	SW	SW	S	S	S	S	S	SE	SE	NE	Tyr
	a	b	c	d	e	f	g	h *	i	j	k	l	m	n	o	p *	q	r
Li	1.69	0.10																
Be	0.017	0.008																0.01
B	12.8	16.6																
F	129 †	1506 †																
Na	6759	11,500	5800					13,900										
Mg	587	2200	1890					3040										
Al	164	3000	193								3.5			190				152
Si	30	680																
P	6147	4400																
K	5776	7100	1050					14,400						10,000				
Ca	381	900	7000					7270										
Sc	0.28	12	0.13															
Ti	2.5	96	24															
V	*0.84*	1.14	0.9											1.69			2.9	5.07
Cr	**2.1**	2.6	1.2	1.3		0.54		1.29						0.78	10	3.5	0.39	0.46
Mn	**13.5**	6.3	8.6		18.7			10.2					12.2	9.5	59	39	3.98	9.19
Fe	**315**	197	329					370			33		441	154		1013	128	149
Co	0.76	0.64	1	0.34										1.27		12.4	0.61	
Ni	2.6	5.2	2.4	3.9		0.85		0.83					32	5.6	16	80	1.38	1.28
Cu	**131**	7.2		10	7.75	3.7	11	9.31		3.6	0.85	4.77	5.58	34	15	16.7	4.6	4.03
Zn	112	308	372	111	154		179	120		121	41	84.7	11.2	90	338	434	138	64.3
Ga	0.12	0.55																
Ge	0.16	1.12																
As	11.9	15.1	14.5	6.4			13				3.2		3.24	9.6		19.9	2.5	31.3
Se	**5.2**	8.8	1.7				2.9						4.55			3.8		2.45
Br	97	61	219				173											
Rb	4.4	3.2	0.8															
Sr	14	35	61														15	
Y	0.17	0.07																
Zr	0.32	0.15																
Nb	0.008	0.019																
Mo	0.87	0.86																17.1
Ru	0.0007	0.0058																
Rh	0.015	0.037																
Pd	0.04	0.53																
Ag	0.65	0.48												0.145				0.01
Cd	**2.4**	3.9		1.4	1.35	0.31		2.77	0.09	0.63	0.59	1.18	0.22	0.9	<0.02		2.6	0.34
In	0.0009	0.017																
Sn	0.12	0.25											0.14	0.34				0.03
Sb	0.036	0.086	0.03															0.01
Te	0.013	0.019																
I	17	14	12															
Cs	0.052	0.036	0.04															
Ba	2.6	3.8	19														4.0	
La	0.54	2.54	0.3															
Ce	0.39	0.14																
Pr	0.053	0.013																
Nd	0.24	0.07																
Sm	0.054	0.014																
Eu	0.013	0.053																
Gd	0.062	0.013																
Tb	0.005	0.022	0.01															
Dy	0.039	0.011																
Ho	0.008	0.002																
Er	0.020	0.018																
Tm	0.0035	0.0030																
Yb	0.018	0.007																
Lu	0.0035	0.0060																
Hf	0.016	0.159																
Ta	0.003	0.012	0															
W	0.048	0.277																
Re	0.0001	0.0011																
Os	0.0012	0.0094																
Ir	0.0002	0.0072																
Pt	0.002	0.050																
Au	0.017	0.278																
Hg	**0.16**	0.96		0.11	0.03		0.02		0.05		<0.05						<0.05	
Tl	0.020	0.016																
Pb	**7.0**	1.7		2.5		0.72		1.10	0.18	3.2	0.43	1.08	0.57	1.3	41	19.9	0.46	0.28
Bi	0.022	0.186																0.01
Th	0.16	0.13	0.08															
U	0.15	0.07	0.2															

a: this work; b: [36]; c: [35]; d: [79]; e: [37], f: [34], g: [38]; h [26]; i: [27]; j: [28]; k: [25]; l: [42]; m: [41]; n: [31]; o: [40]; p: [30]; q: [29]; r: [50]; *: transformed from wet to dry weight basis using the factor 6.29 [80]; †: measured semi-quantitatively.

**Table 3:** Results of the analysis of correlations between the contents of the largest number of elements (*N*) and the functions *F_n_* (1) of the morphometric parameters.

	*n*	*N*	*F_n_*
Pearson’s correlation	1	34	$D^{-1.73}$
	2	45	${\left(DW/W\right)}^{0.74}$
	3	50	${\left(W/DW\right)}^{0.84}{\left(W/H^{4.28}\right)}^{1.53}$
Spearman’s correlation	1	35	$D^{-1.71}$
	2	59	${\left(DW/W\right)}^{0.67}$
	3	63	${\left(DW/W\right)}^{0.73}{\left(H^{3.56}/W\right)}^{0.88}$

## Data Availability

The data presented in this study are available on request from the corresponding author. The data are not publicly available due to the privacy issues.

## Supplementary Materials

The following supporting information can be downloaded at: https://www.mdpi.com/article/10.3390/ani13121950/s1, References [74,75,89,90,102] are cited in the supplementary materials.
