# Supplementary material for: Elements in Soft Tissues of the Young Mediterranean Mussel Mytilus galloprovincialis Lam. 1819 Collected in Sevastopol Bay (Crimea, Black Sea): Effects of Age, Sex, Location, and Principal Morphometric Parameters
Source: Animals (Basel). 2023 Jun 10;13(12):1950. doi: 10.3390/ani13121950 (PMC10295058; doi:10.3390/ani13121950)
Supplement: Supplementary file 1 [file animals-13-01950-s001.zip › animals-2319549-supplementary.pdf]

Supplementary material to the article

Elements in soft tissues of young Mediterranean mussels *Mytilus galloprovincialis* Lam. 1819 collected in Sevastopol Bay (Crimea, Black Sea): Effects of age, sex, location and principal morphometric parameters

authored by

Sergey V. Kapranov, Alexander F. Kozintsev, Nikolay I. Bobko, Vitaliy I. Ryabushko

Simple summary: Mussels accumulate trace elements in their soft tissues to the levels several orders of magnitude higher than in the environment and for this reason are regarded as good trace element pollution bioindicators. Yet, there is little understanding as to which mussel characteristics are most strongly correlated with the element accumulation and on which minimal spatial scale the differences in the accumulation can be observed. We studied the effects of several biological characteristics on contents of 72 elements in soft tissues of mussels sampled from Sevastopol Bay and found that most of the contents decreased with age, which feature was consistent with the decrease in the intercellular water content. There were significant differences in the element accumulation patterns in mussels from different sites of this relatively small water body, and multivariate statistics methods allowed distinguishing individuals sampled from each particular site. Sex differences did not significantly affect the overall element accumulation. Most of elements demonstrated significant correlations with a function of only two gravimetric parameters, namely the ratio of soft tissue dry weight and total weight of the mollusk. The results obtained contribute to understanding the element biochemistry and ageing in mussels and are of interest for improving mussel biomonitoring programmes.

Abstract: Although the mussel *Mytilus galloprovincialis* has been known for decades as an excellent bioindicator of trace element pollution in the marine environment, there is still no information on effects of a suite of its principal morphometric parameters and age on trace element levels in soft tissues. In this work, using inductively coupled plasma mass spectrometry, we studied the contents of 72 elements in soft tissues of *M. galloprovincialis* aged 0.5–4, which was sampled at three stations within a relatively small water body, Sevastopol Bay. Significant effects of age and sampling location on the element contents and soft tissue dry-to-wet weight ratio were discovered. Effects of sex were not significant. It was presumed for the first time that the element content decrease in soft tissues of young mussels can be associated with the decrease in physiological needs for elevated contents of essential elements and intracellular water with age. Combinations of six principal morphometric parameters showed that a function of as few as three parameters (soft tissue dry weight, whole mollusk weight and shell height, with by far the greatest contribution of the dry-to-total weight ratio) formed significant correlations with the contents of the largest possible number of elements (69–88% of the total number). For the first time, it was shown that linear discriminant analysis and canonical analysis of principal coordinates can be successfully used for tracing the exact origin of mussel samples within such a small water area. Canonical analysis of principal coordinates proved to be superior in the correct classification of the samples.

Table S1. Trace element contents in European Reference Material ERM®-CE278k as mean value ± uncertainty at the 95% confidence level, in $\mu g \cdot g^{-1}$ dry weight (d.w.), recoveries as 100 %·Observed / Certified, and relative errors as 100 %·(Observed – Certified) / Certified

	Certified value	Observed value, n = 5	Recovery, %	Relative error, %
As	6.7±0.4	5.7±1.6	85	–15
Cd	0.336±0.025	0.37±0.18	110	10.1
Co	0.21	0.21±0.07	100	0.0
Cr	0.73±0.22	0.85±0.16	116	16
Cu	5.98±0.27	5.60±0.35	93.6	6.35
Fe	161±8	156±3	95.7	3.11
Hg	0.071±0.007	0.067±0.004	94	5.6
Mn	4.88±0.24	4.97±0.61	102	1.84
Ni	0.69±0.15	0.60±0.23	87	–13
Pb	2.18±0.18	2.28±0.55	105	4.59
Rb	2.46±0.16	2.23±0.14	90.7	–9.35
Se	1.62±0.12	1.65±0.34	102	1.85
Sr	19.0±1.2	20.5±4.8	108	7.89
Zn	71±4	65±6	92	–8.5

Table S2. Three-way PERMANOVA applied to Z-standardized element contents in mussels' soft tissues with the Location, Age, and Sex group factors

Source	df	SS	MS	Pseudo-F	p(perm)	Unique perms
Location	2	1316.3	658.13	13.013	0.001	997
Sex	1	104.03	104.03	2.0569	0.063	999
Age	5	765.16	153.03	3.0258	0.001	998
Location×Sex	2	102.7	51.351	1.0153	0.4	998
Location×Age**	5	371.41	74.281	1.4687	0.047	996
Sex×Age	5	386.09	77.218	1.5268	0.057	998
Location×Sex×Age**	5	260.19	52.039	1.0289	0.403	999
Res	94	4754.1	50.575			
Total	119	8568				

** Term has one or more empty cells

Table S3. Pairwise comparisons of the element contents at different mussel ages with graphical insets in case of any significant differences. Tukey's test (diagonally asymmetric tables; Q below and p above the diagonal) is used when the normal distribution hypothesis is not rejected (according to the Shapiro-Wilk test), and Dunn's test (diagonally symmetric tables; p) is run otherwise. Significant ($p<0.05$) and marginal ($p<0.1$) differences are highlighted in pink and gray, respectively. Blue highlights: insignificant differences ($p>0.05$) from the Kruskal-Wallis test. Orange highlights: significantly different dispersions from Levene's test; the Games-Howell pairwise test is applied.

	St. 2	0.5	1	2	3
**Li**	0.5		0.003	0.042	0.005
	1	0.003		0.719	0.871
	2	0.042	0.719		0.646
	3	0.005	0.871	0.646	
**Be**	0.5		0.007	0.104	0.061
	1	4.903		0.976	0.954
	2	3.331	0.582		1
	3	3.681	0.735	0.055	
**B**	0.5		0.365	0.997	1
	1	2.333		0.391	0.463
	2	0.273	2.264		0.992
	3	0.159	2.086	0.406	
**F**	0.5		0.201	1	0.688
	1	2.853		0.278	0.016
	2	0.16	2.582		0.828
	3	1.564	4.467	1.207	
**Na**	0.5		0.809	0.032	0.673
	1	1.262		0.11	0.983
	2	4.08	3.295		0.272
	3	1.599	0.521	2.603	
**Mg**	0.5		0.547	0.014	0.107
	1	1.889		0.115	0.593
	2	4.547	3.264		0.688
	3	3.314	1.783	1.566	
**Al**	0.5		0.016	0.074	0.07
	1	0.016		0.997	0.95
	2	0.074	0.997		0.997
	3	0.07	0.95	0.997	

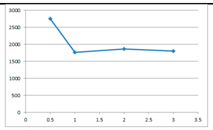
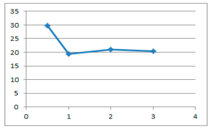
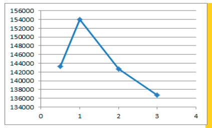
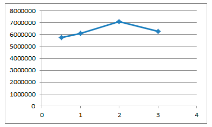
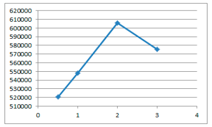
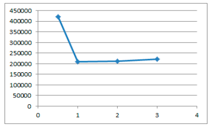

	St. 1	0.5	1	2	3	4
**Li**	0.5		0.145	0.451	0.132	0.721
	1	3.366		0.97	0.999	0.982
	2	2.394	0.882		0.919	1
	3	3.434	0.379	1.176		0.949
	4	1.771	0.773	0.087	1.028	
**Be**	0.5		0.189	0.885	0.253	0.949
	1	3.17		0.719	1	0.868
	2	1.309	1.776		0.759	1
	3	2.937	0.06	1.678		0.877
	4	1.029	1.368	0.012	1.338	
**B**	0.5		0.99	0.976	0.466	0.019
	1	0.664		1	0.727	0.046
	2	0.831	0.185		0.808	0.064
	3	2.359	1.757	1.549		0.405
	4	4.622	4.12	3.915	2.508	
**F**	0.5		0.969	0.365	0.29	0.004
	1	0.969		0.453	0.352	0.008
	2	0.365	0.453		0.988	0.01
	3	0.29	0.352	0.988		0.078
	4	0.004	0.008	0.01	0.078	
**Na**	0.5		1	0.862	0.996	0.868
	1	1		0.862	0.996	0.868
	2	0.862	0.862		0.878	0.767
	3	0.996	0.996	0.878		0.872
	4	0.868	0.868	0.767	0.872	
**Mg**	0.5		0.444	0.086	0.251	0.092
	1	0.444		0.33	0.65	0.269
	2	0.086	0.33		0.657	0.731
	3	0.251	0.65	0.657		0.492
	4	0.092	0.269	0.731	0.492	
**Al**	0.5		0.624	0.955	0.887	1
	1	1.998		0.254	0.996	0.895
	2	0.989	2.934		0.546	0.955
	3	1.302	0.512	2.175		0.979
	4	0.238	1.273	0.99	0.799	

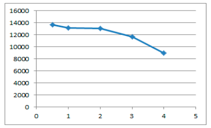
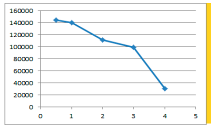

	St. 3	0.5	1	1.5	2
**Li**	0.5		0.071	0.659	0.595
	1	0.071		0.2	0.301
	1.5	0.659	0.2		0.897
	2	0.595	0.301	0.897	
**Be**	0.5		0.12	0.388	0.697
	1	0.12		0.516	0.339
	1.5	0.388	0.516		0.714
	2	0.697	0.339	0.714	
**B**	0.5		0.271	0.019	0.023
	1	2.604		2E-4	5E-4
	1.5	4.388	6.576		0.996
	2	4.264	6.278	0.31	
**F**	0.5		0.718	0.981	0.807
	1	0.718		0.202	0.03
	1.5	0.981	0.202		0.893
	2	0.807	0.03	0.893	
**Na**	0.5		0.553	0.054	0.04
	1	1.875		0.003	0.003
	1.5	3.765	5.305		0.981
	2	3.949	5.357	0.543	
**Mg**	0.5		0.466	0.075	0.044
	1	2.078		0.003	0.002
	1.5	3.55	5.293		0.966
	2	3.88	5.465	0.661	
**Al**	0.5		0.227	0.846	0.919
	1	0.227		0.187	0.35
	1.5	0.846	0.187		0.793
	2	0.919	0.35	0.793	

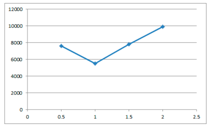
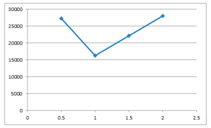
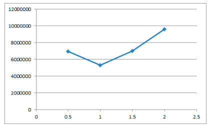
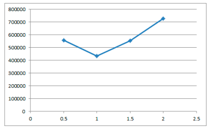

Table S3. Continued

	St. 2	0.5	1	2	3	
**Si**	0.5		0.201	0.183	0.289	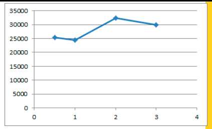
	1	0.201		0.012	0.017	
	2	0.183	0.012		0.705	
	3	0.289	0.017	0.705		
**P**	0.5		0.037	0.139	0.392	
	1	0.037		0.859	0.279	
	2	0.139	0.859		0.482	
	3	0.392	0.279	0.482		
**K**	0.5		0.029	0.476	0.532	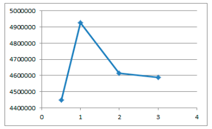
	1	0.029		0.280	0.153	
	2	0.476	0.280		0.878	
	3	0.532	0.153	0.878		
**Ca**	0.5		0.398	0.354	0.302	
	1	0.398		0.781	0.759	
	2	0.354	0.781		0.993	
	3	0.302	0.759	0.993		
**Sc**	0.5		0.455	0.566	0.436	
	1	0.455		0.986	0.900	
	2	0.566	0.986		0.907	
	3	0.436	0.900	0.907		
**Ti**	0.5		0.047	0.075	0.008	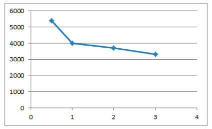
	1	0.047		0.827	0.326	
	2	0.075	0.827		0.558	
	3	0.008	0.326	0.558		
**V**	0.5		0.003	0.005	0.002	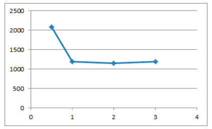
	1	0.003		0.633	0.639	
	2	0.005	0.633		0.950	
	3	0.002	0.639	0.950		

	St. 1	0.5	1	2	3	4	
**Si**	0.5		0.814	0.608	0.600	0.007	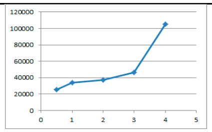
	1	0.814		0.998	0.937	0.003	
	2	0.608	0.998		0.980	0.003	
	3	0.600	0.937	0.980		0.031	
	4	0.007	0.003	0.003	0.031		
**P**	0.5		0.221	0.005	0.539	0.675	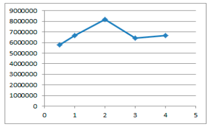
	1	0.221		0.107	0.620	0.613	
	2	0.005	0.107		0.050	0.083	
	3	0.539	0.620	0.050		0.930	
	4	0.675	0.613	0.083	0.930		
**K**	0.5		1.000	0.063	0.031	0.003	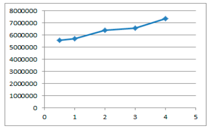
	1	1.000		0.063	0.031	0.003	
	2	0.063	0.063		0.680	0.126	
	3	0.031	0.031	0.680		0.256	
	4	0.003	0.003	0.126	0.256		
**Ca**	0.5		0.706	0.931	0.704	0.910	
	1	1.808		0.991	1.000	0.377	
	2	1.120	0.640		0.984	0.601	
	3	1.812	0.171	0.751		0.381	
	4	1.212	2.579	2.050	2.569		
**Sc**	0.5		0.284	0.742	0.477	0.902	
	1	0.284		0.475	0.795	0.492	
	2	0.742	0.475		0.692	0.896	
	3	0.477	0.795	0.692		0.657	
	4	0.902	0.492	0.896	0.657		
**Ti**	0.5		0.528	0.433	0.126	0.063	
	1	0.528		0.865	0.339	0.167	
	2	0.433	0.865		0.435	0.219	
	3	0.126	0.339	0.435		0.582	
	4	0.063	0.167	0.219	0.582		
**V**	0.5		0.221	0.062	0.102	0.014	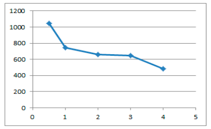
	1	0.221		0.499	0.601	0.127	
	2	0.062	0.499		0.916	0.325	
	3	0.102	0.601	0.916		0.304	
	4	0.014	0.127	0.325	0.304		

	St. 3	0.5	1	1.5	2	
**Si**	0.5		0.011	0.713	0.618	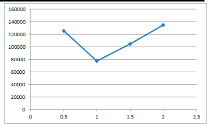
	1	0.011		0.006	0.008	
	1.5	0.713	0.006		0.872	
	2	0.618	0.008	0.872		
**P**	0.5		0.677	0.009	0.042	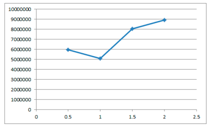
	1	0.677		0.004	0.022	
	1.5	0.009	0.004		0.768	
	2	0.042	0.022	0.768		
**K**	0.5		0.184	0.085	0.123	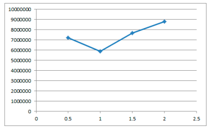
	1	0.184		0.004	0.009	
	1.5	0.085	0.004		1.000	
	2	0.123	0.009	1.000		
**Ca**	0.5		0.017	0.529	0.557	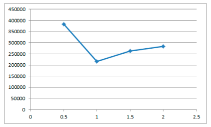
	1	4.429		0.359	0.496	
	1.5	1.931	2.349		1.000	
	2	1.865	2.006	0.126		
**Sc**	0.5		0.961	0.357	0.656	
	1	0.695		0.197	0.435	
	1.5	2.354	2.868		0.989	
	2	1.639	2.153	0.450		
**Ti**	0.5		0.030	0.226	0.117	
	1	0.030		0.369	0.719	
	1.5	0.226	0.369		0.648	
	2	0.117	0.719	0.648		
**V**	0.5		0.061	0.382	0.450	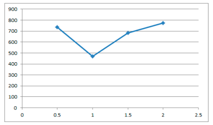
	1	0.061		0.010	0.021	
	1.5	0.382	0.010		0.978	
	2	0.450	0.021	0.978		

Table S3. Continued

	St. 2	0.5	1	2	3	
**Cr**	0.5		0.004	0.002	0.001	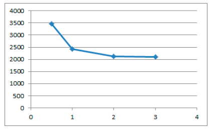
	1	5.222		0.718	0.620	
	2	5.525	1.493		1.000	
	3	6.219	1.721	0.009		
**Mn**	0.5		0.000	0.007	0.009	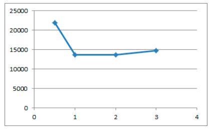
	1	0.000		0.901	0.546	
	2	0.007	0.901		0.712	
	3	0.009	0.546	0.712		
**Fe**	0.5		0.006	0.020	0.014	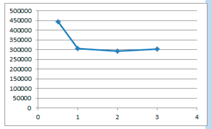
	1	0.006		0.883	1.000	
	2	0.020	0.883		0.892	
	3	0.014	1.000	0.892		
**Co**	0.5		0.034	0.118	0.248	
	1	0.034		0.906	0.430	
	2	0.118	0.906		0.601	
	3	0.248	0.430	0.601		
**Ni**	0.5		0.000	0.000	0.000	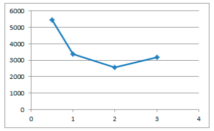
	1	6.853		0.400	0.999	
	2	7.514	2.241		0.550	
	3	6.286	0.214	1.882		
**Cu**	0.5		1.000	0.890	0.409	
	1	0.108		0.896	0.373	
	2	1.012	0.990		0.909	
	3	2.217	2.312	0.942		
**Zn**	0.5		0.013	0.024	0.020	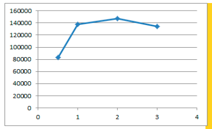
	1	0.013		0.754	0.903	
	2	0.024	0.754		0.850	
	3	0.020	0.903	0.850		

	St. 1	0.5	1	2	3	4	
**Cr**	0.5		0.528	0.865	0.014	0.009	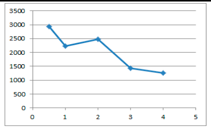
	1	0.528		0.657	0.061	0.033	
	2	0.865	0.657		0.025	0.015	
	3	0.014	0.061	0.025		0.589	
	4	0.009	0.033	0.015	0.589		
**Mn**	0.5		0.917	1.000	0.930	0.655	
	1	1.184		0.909	1.000	0.948	
	2	0.064	1.216		0.923	0.647	
	3	1.125	0.051	1.159		0.964	
	4	1.926	1.031	1.945	0.933		
**Fe**	0.5		0.671	0.995	0.967	0.338	
	1	1.890		0.890	0.978	0.046	
	2	0.548	1.291		0.999	0.217	
	3	0.907	0.808	0.387		0.171	
	4	2.683	4.112	3.061	3.246		
**Co**	0.5		0.349	0.432	0.528	0.085	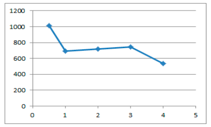
	1	0.349		0.995	0.953	0.073	
	2	0.432	0.995		0.997	0.055	
	3	0.528	0.953	0.997		0.070	
	4	0.085	0.073	0.055	0.070		
**Ni**	0.5		0.740	0.272	0.175	0.116	
	1	0.740		0.603	0.340	0.271	
	2	0.272	0.603		0.990	0.804	
	3	0.175	0.340	0.990		0.942	
	4	0.116	0.271	0.804	0.942		
**Cu**	0.5		0.000	0.007	0.611	0.772	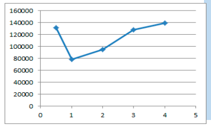
	1	0.000		0.316	0.003	0.009	
	2	0.007	0.316		0.047	0.073	
	3	0.611	0.003	0.047		0.899	
	4	0.772	0.009	0.073	0.899		
**Zn**	0.5		0.118	0.928	0.995	1.000	
	1	0.118		0.805	0.514	0.298	
	2	0.928	0.805		0.905	0.921	
	3	0.995	0.514	0.905		0.998	
	4	1.000	0.298	0.921	0.998		

	St. 3	0.5	1	1.5	2	
**Cr**	0.5		0.741	0.486	0.226	
	1	0.741		0.731	0.384	
	1.5	0.486	0.731		0.577	
	2	0.226	0.384	0.577		
**Mn**	0.5		0.584	0.328	0.182	
	1	0.584		0.151	0.083	
	1.5	0.328	0.151		0.664	
	2	0.182	0.083	0.664		
**Fe**	0.5		0.022	0.878	0.717	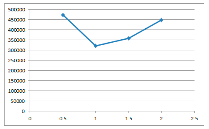
	1	0.022		0.045	0.022	
	1.5	0.878	0.045		0.634	
	2	0.717	0.022	0.634		
**Co**	0.5		0.900	0.022	0.021	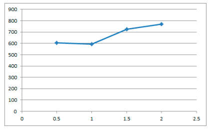
	1	0.900		0.023	0.021	
	1.5	0.022	0.023		0.810	
	2	0.021	0.021	0.810		
**Ni**	0.5		0.237	0.231	0.366	
	1	0.237		0.025	0.062	
	1.5	0.231	0.025		0.872	
	2	0.366	0.062	0.872		
**Cu**	0.5		0.077	0.031	0.081	
	1	0.077		0.716	0.880	
	1.5	0.031	0.716		0.858	
	2	0.081	0.880	0.858		
**Zn**	0.5		0.794	0.087	0.165	
	1	0.794		0.064	0.122	
	1.5	0.087	0.064		0.890	
	2	0.165	0.122	0.890		

Table S3. Continued

	St. 2	0.5	1	2	3	
**Ga**	0.5		0.001	0.017	0.001	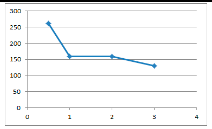
	1	5.709		1.000	0.898	
	2	4.445	0.073		0.930	
	3	5.974	0.983	0.853		
**Ge**	0.5		0.080	0.071	0.030	
	1	0.080		0.654	0.502	
	2	0.071	0.654		0.900	
	3	0.030	0.502	0.900		
**As**	0.5		0.393	0.550	0.139	
	1	2.258		1.000	0.821	
	2	1.881	0.102		0.922	
	3	3.133	1.227	0.888		
**Se**	0.5		0.887	0.943	0.917	
	1	1.025		0.623	0.506	
	2	0.794	1.715		1.000	
	3	0.910	1.984	0.015		
**Br**	0.5		0.762	0.311	0.006	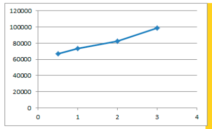
	1	1.386		0.722	0.030	
	2	2.484	1.484		0.531	
	3	5.001	4.107	1.926		
**Rb**	0.5		0.398	0.115	0.104	
	1	0.398		0.397	0.302	
	2	0.115	0.397		1.000	
	3	0.104	0.302	1.000		
**Sr**	0.5		0.349	0.655	0.180	
	1	0.349		0.754	0.579	
	2	0.655	0.754		0.465	
	3	0.180	0.579	0.465		

	St. 1	0.5	1	2	3	4	
**Ga**	0.5		0.440	0.882	0.782	0.344	
	1	2.422		0.947	0.994	0.976	
	2	1.320	1.037		0.999	0.783	
	3	1.618	0.580	0.379		0.903	
	4	2.665	0.834	1.615	1.244		
**Ge**	0.5		0.449	0.401	0.258	0.594	
	1	2.400		1.000	0.984	1.000	
	2	2.517	0.182		0.995	1.000	
	3	2.921	0.744	0.561		0.999	
	4	2.065	0.251	0.108	0.348		
**As**	0.5		1.000	1.000	0.636	0.600	
	1	0.118		1.000	0.683	0.639	
	2	0.088	0.203		0.620	0.584	
	3	1.969	1.862	2.006		0.999	
	4	2.053	1.964	2.088	0.390		
**Se**	0.5		0.760	0.029	0.021	0.001	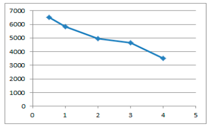
	1	0.760		0.059	0.042	0.002	
	2	0.029	0.059		0.788	0.103	
	3	0.021	0.042	0.788		0.179	
	4	0.001	0.002	0.103	0.179		
**Br**	0.5		0.984	0.205	0.476	0.999	
	1	0.984		0.057	0.318	0.947	
	2	0.205	0.057		0.999	0.525	
	3	0.476	0.318	0.999		0.652	
	4	0.999	0.947	0.525	0.652		
**Rb**	0.5		0.807	0.844	0.276	0.002	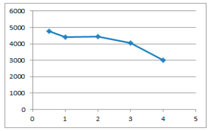
	1	1.551		1.000	0.840	0.020	
	2	1.444	0.065		0.831	0.021	
	3	2.863	1.455	1.482		0.183	
	4	5.769	4.596	4.575	3.194		
**Sr**	0.5		0.950	1.000	1.000	0.759	
	1	1.020		0.940	0.987	0.428	
	2	0.086	1.079		1.000	0.794	
	3	0.217	0.709	0.290		0.728	
	4	1.678	2.449	1.587	1.754		

	St. 3	0.5	1	1.5	2	
**Ga**	0.5		0.242	0.295	0.601	
	1	0.242		0.037	0.616	
	1.5	0.295	0.037		0.164	
	2	0.601	0.616	0.164		
**Ge**	0.5		0.120	0.176	0.021	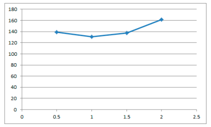
	1	0.120		0.006	0.000	
	1.5	0.176	0.006		0.302	
	2	0.021	0.000	0.302		
**As**	0.5		0.993	0.644	0.392	
	1	0.390		0.528	0.311	
	1.5	1.666	1.934		0.955	
	2	2.261	2.484	0.729		
**Se**	0.5		0.896	0.433	0.054	
	1	0.896		0.389	0.051	
	1.5	0.433	0.389		0.243	
	2	0.054	0.051	0.243		
**Br**	0.5		0.032	0.783	0.321	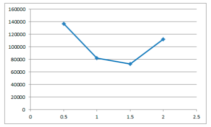
	1	0.032		0.023	0.006	
	1.5	0.783	0.023		0.478	
	2	0.321	0.006	0.478		
**Rb**	0.5		0.775	0.005	0.037	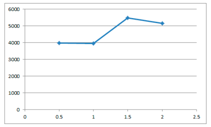
	1	0.775		0.018	0.081	
	1.5	0.005	0.018		0.682	
	2	0.037	0.081	0.682		
**Sr**	0.5		0.005	0.936	0.712	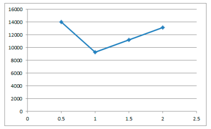
	1	0.005		0.010	0.006	
	1.5	0.936	0.010		0.675	
	2	0.712	0.006	0.675		

Table S3. Continued

	St. 2	0.5	1	2	3
**Y**	0.5		0.137	0.151	0.809
	1	0.137		1.000	0.822
	2	0.151	1.000		0.807
	3	0.809	0.822	0.807	
**Zr**	0.5		0.004	0.249	0.010
	1	0.004		0.235	0.960
	2	0.249	0.235		0.260
	3	0.010	0.960	0.260	
**Nb**	0.5		0.479	0.471	0.465
	1	0.479		0.999	0.994
	2	0.471	0.999		1.000
	3	0.465	0.994	1.000	
**Mo**	0.5		0.077	0.213	0.003
	1	0.077		0.955	0.018
	2	0.213	0.955		0.727
	3	0.003	0.018	0.727	
**Ru**	0.5		0.476	0.457	0.468
	1	0.476		0.054	0.939
	2	0.457	0.054		0.864
	3	0.468	0.939	0.864	
**Rh**	0.5		0.390	0.058	0.003
	1	0.390		0.192	0.018
	2	0.058	0.192		0.488
	3	0.003	0.018	0.488	
**Pd**	0.5		0.044	0.026	0.002
	1	0.044		0.494	0.171
	2	0.026	0.494		0.639
	3	0.002	0.171	0.639	

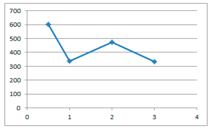
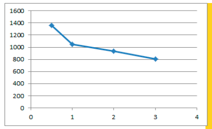
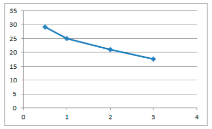
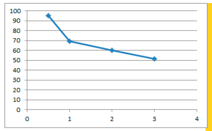

	St. 1	0.5	1	2	3	4
**Y**	0.5		0.718	0.985	0.728	1.000
	1	1.779		0.953	1.000	0.865
	2	0.730	1.002		0.945	0.993
	3	1.756	0.141	1.052		0.855
	4	0.032	1.377	0.589	1.410	
**Zr**	0.5		0.660	0.184	0.730	0.795
	1	0.660		0.079	0.457	0.942
	2	0.184	0.079		0.381	0.203
	3	0.730	0.457	0.381		0.605
	4	0.795	0.942	0.203	0.605	
**Nb**	0.5		0.847	0.899	0.994	0.921
	1	0.847		0.797	0.606	0.005
	2	0.899	0.797		0.790	0.965
	3	0.994	0.606	0.790		0.854
	4	0.921	0.005	0.965	0.854	
**Mo**	0.5		0.999	0.977	1.000	0.390
	1	0.999		0.689	1.000	0.024
	2	0.977	0.689		0.851	0.022
	3	1.000	1.000	0.851		0.253
	4	0.390	0.024	0.022	0.253	
**Ru**	0.5		0.818	0.911	0.992	0.990
	1	0.818		0.510	0.571	0.004
	2	0.911	0.510		0.876	0.108
	3	0.992	0.571	0.876		1.000
	4	0.990	0.004	0.108	1.000	
**Rh**	0.5		0.038	0.171	0.436	0.147
	1	4.229		0.971	0.856	1.000
	2	3.243	0.874		0.994	0.977
	3	2.432	1.406	0.579		0.899
	4	3.357	0.160	0.825	1.256	
**Pd**	0.5		0.067	0.407	0.915	0.966
	1	0.067		0.356	0.680	0.366
	2	0.407	0.356		0.778	0.858
	3	0.915	0.680	0.778		0.863
	4	0.966	0.366	0.858	0.863	

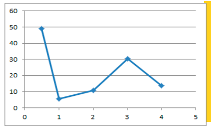
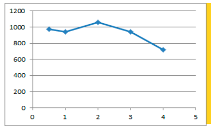
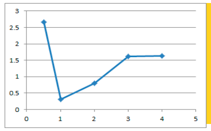
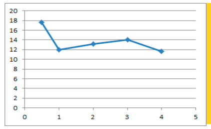

	St. 3	0.5	1	1.5	2
**Y**	0.5		0.554	0.350	0.811
	1	0.554		0.151	0.781
	1.5	0.350	0.151		0.306
	2	0.811	0.781	0.306	
**Zr**	0.5		0.287	0.534	0.598
	1	0.287		0.112	0.158
	1.5	0.534	0.112		0.976
	2	0.598	0.158	0.976	
**Nb**	0.5		0.444	0.766	0.545
	1	0.444		0.375	0.631
	1.5	0.766	0.375		0.411
	2	0.545	0.631	0.411	
**Mo**	0.5		0.137	0.295	0.276
	1	0.137		0.279	0.657
	1.5	0.295	0.279		0.986
	2	0.276	0.657	0.986	
**Ru**	0.5		0.862	0.626	0.993
	1	0.862		0.548	0.454
	1.5	0.626	0.548		0.649
	2	0.993	0.454	0.649	
**Rh**	0.5		0.991	0.692	0.958
	1	0.991		0.649	0.997
	1.5	0.692	0.649		0.629
	2	0.958	0.997	0.629	
**Pd**	0.5		0.766	0.720	0.987
	1	0.766		0.538	0.843
	1.5	0.720	0.538		0.644
	2	0.987	0.843	0.644	

Table S3. Continued

	St. 2	0.5	1	2	3		St. 1	0.5	1	2	3	4	St. 3	0.5	1	1.5	2
**Ag**	0.5		0.726	0.445	0.608	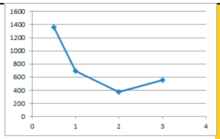	0.5		0.402	0.992	0.849	0.691	0.5		0.999	0.869	0.192
	1	0.726		0.002	0.706		1	0.402		0.798	0.798	0.623	1	0.999		0.908	0.770
	2	0.445	0.002		0.519		2	0.992	0.798		0.884	0.747	1.5	0.869	0.908		0.295
	3	0.608	0.706	0.519			3	0.849	0.798	0.884		1.000	2	0.192	0.770	0.295	
							4	0.691	0.623	0.747	1.000						
**Cd**	0.5		0.383	0.180	0.132		0.5		0.433	0.879	0.927	0.357	0.5		0.252	0.520	0.572
	1	0.383		0.486	0.425		1	0.433		0.701	0.836	0.232	1	0.252		0.981	0.961
	2	0.180	0.486		1.000		2	0.879	0.701		1.000	0.277	1.5	0.520	0.981		1.000
	3	0.132	0.425	1.000			3	0.927	0.836	1.000		0.280	2	0.572	0.961	1.000	
							4	0.357	0.232	0.277	0.280						
**In**	0.5		0.156	0.150	0.196		0.5		0.135	0.896	0.911	0.977	0.5		0.315	0.849	0.568
	1	0.156		0.996	0.817		1	0.135		0.562	0.672	0.502	1	0.315		0.495	0.418
	2	0.150	0.996		0.766		2	0.896	0.562		1.000	0.822	1.5	0.849	0.495		0.584
	3	0.196	0.817	0.766			3	0.911	0.672	1.000		0.829	2	0.568	0.418	0.584	
							4	0.977	0.502	0.822	0.829						
**Sn**	0.5		0.005	0.009	0.008	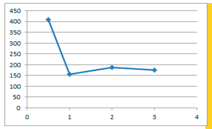	0.5		0.581	0.901	1.000	1.000	0.5		0.494	0.883	0.575
	1	0.005		0.850	0.916		1	0.581		0.835	0.469	0.492	1	0.494		0.382	0.729
	2	0.009	0.850		1.000		2	0.901	0.835		0.812	0.891	1.5	0.883	0.382		0.421
	3	0.008	0.916	1.000			3	1.000	0.469	0.812		0.997	2	0.575	0.729	0.421	
							4	1.000	0.492	0.891	0.997						
**Sb**	0.5		0.521	0.001	0.041	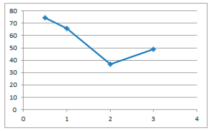	0.5		0.293	0.235	0.652	0.811	0.5		0.264	0.622	0.761
	1	0.521		0.004	0.108		1	0.293		0.870	0.615	0.301	1	0.264		0.616	0.694
	2	0.001	0.004		0.185		2	0.235	0.870		0.521	0.253	1.5	0.622	0.616		0.618
	3	0.041	0.108	0.185			3	0.652	0.615	0.521		0.562	2	0.761	0.694	0.618	
							4	0.811	0.301	0.253	0.562						
**Te**	0.5		0.363	0.327	0.360		0.5		0.855	0.859	0.877	0.838	0.5		0.425	0.601	0.653
	1	0.363		0.727	0.999		1	0.855		0.998	0.903	0.799	1	0.425		0.411	0.978
	2	0.327	0.727		0.945		2	0.859	0.998		0.956	0.699	1.5	0.601	0.411		0.442
	3	0.360	0.999	0.945			3	0.877	0.903	0.956		0.625	2	0.653	0.978	0.442	
							4	0.838	0.799	0.699	0.625						
**I**	0.5		0.000	0.012	0.001	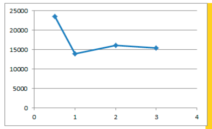	0.5		0.307	0.215	0.585	0.361	0.5		0.075	0.792	0.986
	1	0.000		0.302	0.513		1	0.307		0.934	0.995	1.000	1	0.075		0.118	0.747
	2	0.012	0.302		0.672		2	0.215	0.934		0.929	0.917	1.5	0.792	0.118		0.928
	3	0.001	0.513	0.672			3	0.585	0.995	0.929		0.999	2	0.986	0.747	0.928	
							4	0.361	1.000	0.917	0.999						

Table S3. Continued

	St. 2	0.5	1	2	3	St. 1	0.5	1	2	3	4	St. 3	0.5	1	1.5	2
**Cs**	0.5		0.005	0.019	0.019	0.5		0.468	0.443	0.365	0.627	0.5		0.690	0.760	0.940
	1	5.060		0.991	1.000	1	2.354		1.000	0.997	1.000	1	1.561		1.000	0.978
	2	4.385	0.412		0.985	2	2.415	0.124		0.999	1.000	1.5	1.390	0.161		0.991
	3	4.363	0.144	0.493		3	2.609	0.473	0.349		1.000	2	0.809	0.563	0.417	
						4	1.990	0.211	0.113	0.173						
**Ba**	0.5		0.460	0.280	0.335	0.5		0.228	0.529	0.484	0.931	0.5		0.413	0.300	0.997
	1	2.092		0.898	0.973	1	0.228		0.586	0.693	0.410	1	2.208		0.997	0.672
	2	2.575	0.986		0.991	2	0.529	0.586		0.912	0.693	1.5	2.516	0.290		0.557
	3	2.413	0.605	0.420		3	0.484	0.693	0.912		0.640	2	0.296	1.603	1.866	
						4	0.931	0.410	0.693	0.640						
**La**	0.5		0.144	0.411	0.043	0.5		0.745	0.798	0.059	0.506	0.5		0.515	0.524	0.986
	1	0.144		0.723	0.430	1	0.745		0.952	0.111	0.675	1	0.515		1.000	0.723
	2	0.411	0.723		0.339	2	0.798	0.952		0.107	0.647	1.5	0.524	1.000		0.734
	3	0.043	0.430	0.339		3	0.059	0.111	0.107		0.391	2	0.986	0.723	0.734	
						4	0.506	0.675	0.647	0.391						
**Ce**	0.5		0.032	0.056	0.029	0.5		0.252	0.627	0.498	0.992	0.5		0.109	0.545	0.368
	1	0.032		0.818	0.766	1	2.941		0.972	0.999	0.792	1	0.109		0.349	0.610
	2	0.056	0.818		0.978	2	1.992	0.871		0.998	0.968	1.5	0.545	0.349		0.734
	3	0.029	0.766	0.978		3	2.284	0.385	0.418		0.913	2	0.368	0.610	0.734	
						4	0.630	1.593	0.903	1.201						
**Pr**	0.5		0.036	0.053	0.069	0.5		0.066	0.288	0.129	0.868	0.5		0.003	0.345	0.586
	1	0.036		0.763	0.960	1	0.066		0.469	0.882	0.120	1	0.003		0.056	0.043
	2	0.053	0.763		0.752	2	0.288	0.469		0.607	0.329	1.5	0.345	0.056		0.774
	3	0.069	0.960	0.752		3	0.129	0.882	0.607		0.177	2	0.586	0.043	0.774	
						4	0.868	0.120	0.329	0.177						
**Nd**	0.5		0.096	0.267	0.155	0.5		0.149	0.235	0.067	0.491	0.5		0.074	0.489	0.639
	1	0.096		0.827	0.953	1	3.344		1.000	0.974	1.000	1	0.074		0.302	0.280
	2	0.267	0.827		0.878	2	2.996	0.259		0.942	1.000	1.5	0.489	0.302		0.886
	3	0.155	0.953	0.878		3	3.885	0.851	1.068		0.970	2	0.639	0.280	0.886	
						4	2.301	0.227	0.025	0.883						
**Sm**	0.5		0.301	0.639	0.311	0.5		0.113	0.500	0.668	0.986	0.5		0.030	0.626	0.815
	1	0.301		0.710	0.917	1	3.540		0.921	0.883	0.139	1	0.030		0.112	0.102
	2	0.639	0.710		0.672	2	2.280	1.166		1.000	0.428	1.5	0.626	0.112		0.847
	3	0.311	0.917	0.672		3	1.897	1.316	0.224		0.548	2	0.815	0.102	0.847	
						4	0.719	3.395	2.451	2.170						

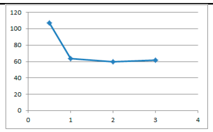

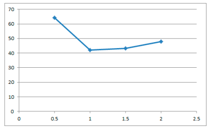

Table S3. Continued

	St. 2	0.5	1	2	3
**Eu**	0.5		0.135	0.251	0.608
	1	3.149		0.999	0.850
	2	2.670	0.193		0.867
	3	1.750	1.142	1.090	
**Gd**	0.5		0.422	0.791	0.445
	1	0.422		0.693	0.957
	2	0.791	0.693		0.685
	3	0.445	0.957	0.685	
**Tb**	0.5		0.005	0.014	0.010
	1	0.005		0.804	0.953
	2	0.014	0.804		0.857
	3	0.010	0.953	0.857	
**Dy**	0.5		0.096	0.302	0.563
	1	3.391		0.999	0.807
	2	2.508	0.184		0.932
	3	1.851	1.266	0.844	
**Ho**	0.5		0.241	0.371	0.831
	1	2.706		0.999	0.790
	2	2.317	0.190		0.823
	3	1.200	1.312	1.224	
**Er**	0.5		0.144	0.321	0.825
	1	3.103		1.000	0.639
	2	2.453	0.001		0.778
	3	1.216	1.679	1.343	
**Tm**	0.5		0.234	0.219	0.928
	1	0.234		0.959	0.927
	2	0.219	0.959		0.821
	3	0.928	0.927	0.821	

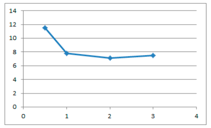

	St. 1	0.5	1	2	3	4
**Eu**	0.5		0.331	0.916	0.822	0.006
	1	0.331		0.314	0.275	0.007
	2	0.916	0.314		0.999	0.011
	3	0.822	0.275	0.999		0.023
	4	0.006	0.007	0.011	0.023	
**Gd**	0.5		0.031	0.366	0.074	0.908
	1	0.031		0.230	0.862	0.129
	2	0.366	0.230		0.355	0.564
	3	0.074	0.862	0.355		0.195
	4	0.908	0.129	0.564	0.195	
**Tb**	0.5		0.293	0.534	0.206	0.938
	1	2.812		0.996	0.995	0.947
	2	2.203	0.534		0.949	0.992
	3	3.102	0.550	1.025		0.855
	4	1.089	1.037	0.612	1.411	
**Dy**	0.5		0.195	0.965	0.816	0.391
	1	3.144		0.561	0.880	0.011
	2	0.921	2.139		0.990	0.180
	3	1.527	1.326	0.654		0.103
	4	2.542	4.919	3.207	3.600	
**Ho**	0.5		0.403	0.774	0.663	0.004
	1	0.403		0.374	0.333	0.005
	2	0.774	0.374		0.999	0.006
	3	0.663	0.333	0.999		0.020
	4	0.004	0.005	0.006	0.020	
**Er**	0.5		0.082	0.939	0.923	0.034
	1	0.082		0.106	0.094	0.001
	2	0.939	0.106		0.870	0.031
	3	0.923	0.094	0.870		0.054
	4	0.034	0.001	0.031	0.054	
**Tm**	0.5		0.509	0.564	0.492	0.003
	1	0.509		0.408	0.382	0.004
	2	0.564	0.408		0.997	0.007
	3	0.492	0.382	0.997		0.045
	4	0.003	0.004	0.007	0.045	

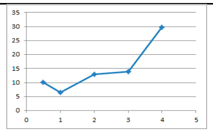
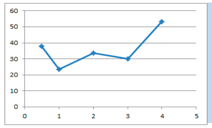
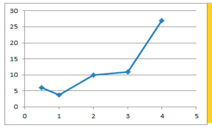
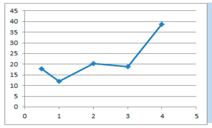
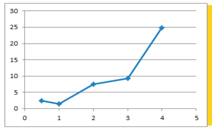

	St. 3	0.5	1	1.5	2
**Eu**	0.5		0.008	0.802	0.370
	1	0.008		0.023	0.002
	1.5	0.802	0.023		0.286
	2	0.370	0.002	0.286	
**Gd**	0.5		0.049	0.452	0.876
	1	0.049		0.251	0.125
	1.5	0.452	0.251		0.622
	2	0.876	0.125	0.622	
**Tb**	0.5		0.019	0.302	0.666
	1	0.019		0.214	0.110
	1.5	0.302	0.214		0.638
	2	0.666	0.110	0.638	
**Dy**	0.5		0.019	0.443	0.650
	1	0.019		0.136	0.115
	1.5	0.443	0.136		0.823
	2	0.650	0.115	0.823	
**Ho**	0.5		0.017	0.913	0.987
	1	0.017		0.045	0.003
	1.5	0.913	0.045		0.634
	2	0.987	0.003	0.634	
**Er**	0.5		0.024	0.155	0.309
	1	0.024		0.433	0.337
	1.5	0.155	0.433		0.804
	2	0.309	0.337	0.804	
**Tm**	0.5		0.051	0.979	0.926
	1	0.051		0.038	0.003
	1.5	0.979	0.038		0.570
	2	0.926	0.003	0.570	

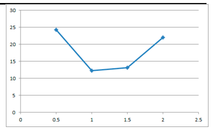
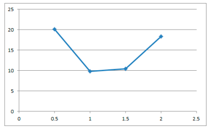
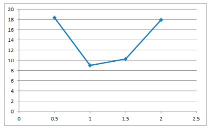

Table S3. Continued

	St. 2	0.5	1	2	3	
**Yb**	0.5		0.025	0.120	0.348	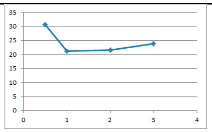
	1	4.215		1.000	0.720	
	2	3.237	0.101		0.865	
	3	2.379	1.488	1.098		
**Lu**	0.5		0.009	0.022	0.035	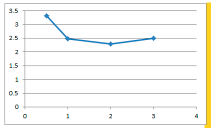
	1	0.009		0.813	0.822	
	2	0.022	0.813		0.692	
	3	0.035	0.822	0.692		
**Hf**	0.5		0.091	0.056	0.066	
	1	0.091		0.059	0.488	
	2	0.056	0.059		0.867	
	3	0.066	0.488	0.867		
**Ta**	0.5		0.680	0.365	0.391	
	1	0.680		0.308	0.396	
	2	0.365	0.308		0.368	
	3	0.391	0.396	0.368		
**W**	0.5		0.347	0.983	0.108	
	1	0.347		0.956	0.089	
	2	0.983	0.956		0.798	
	3	0.108	0.089	0.798		
**Re**	0.5		0.002	0.007	0.009	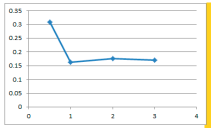
	1	0.002		0.836	0.839	
	2	0.007	0.836		0.725	
	3	0.009	0.839	0.725		
**Os**	0.5		0.126	0.088	0.097	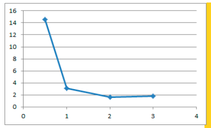
	1	0.126		0.017	0.329	
	2	0.088	0.017		0.889	
	3	0.097	0.329	0.889		

	St. 1	0.5	1	2	3	4	
**Yb**	0.5		0.251	0.899	0.489	1.000	
	1	2.942		0.786	0.999	0.464	
	2	1.256	1.608		0.933	0.936	
	3	2.306	0.364	1.110		0.647	
	4	0.139	2.363	1.097	1.945		
**Lu**	0.5		0.523	0.563	0.496	0.003	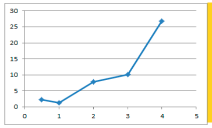
	1	0.523		0.414	0.393	0.004	
	2	0.563	0.414		0.996	0.007	
	3	0.496	0.393	0.996		0.054	
	4	0.003	0.004	0.007	0.054		
**Hf**	0.5		0.869	1.000	0.872	0.946	
	1	0.869		0.853	0.647	0.307	
	2	1.000	0.853		0.830	0.849	
	3	0.872	0.647	0.830		0.968	
	4	0.946	0.307	0.849	0.968		
**Ta**	0.5		0.851	0.912	0.998	0.980	
	1	0.851		0.762	0.433	0.771	
	2	0.912	0.762		0.667	0.809	
	3	0.998	0.433	0.667		0.926	
	4	0.980	0.771	0.809	0.926		
**W**	0.5		0.521	0.749	0.605	0.994	
	1	0.521		0.970	0.996	0.556	
	2	0.749	0.970		0.997	0.675	
	3	0.605	0.996	0.997		0.596	
	4	0.994	0.556	0.675	0.596		
**Re**	0.5		0.156	0.446	0.996	0.591	
	1	0.156		0.932	0.856	0.997	
	2	0.446	0.932		0.906	1.000	
	3	0.996	0.856	0.906		0.900	
	4	0.591	0.997	1.000	0.900		
**Os**	0.5		0.031	0.828	0.489	0.592	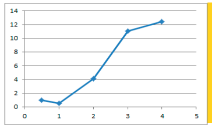
	1	0.031		0.757	0.455	0.567	
	2	0.828	0.757		0.825	0.820	
	3	0.489	0.455	0.825		1.000	
	4	0.592	0.567	0.820	1.000		

	St. 3	0.5	1	1.5	2	
**Yb**	0.5		0.174	0.381	0.143	
	1	0.174		0.035	0.011	
	1.5	0.381	0.035		0.519	
	2	0.143	0.011	0.519		
**Lu**	0.5		0.124	0.984	0.919	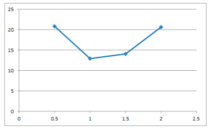
	1	0.124		0.060	0.003	
	1.5	0.984	0.060		0.508	
	2	0.919	0.003	0.508		
**Hf**	0.5		0.016	0.002	0.999	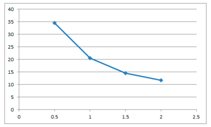
	1	0.016		0.836	0.921	
	1.5	0.002	0.836		0.854	
	2	0.999	0.921	0.854		
**Ta**	0.5		0.003	0.002	0.823	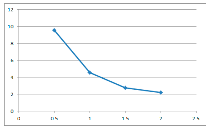
	1	0.003		0.010	0.777	
	1.5	0.002	0.010		0.762	
	2	0.823	0.777	0.762		
**W**	0.5		0.113	0.002	0.012	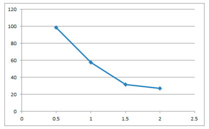
	1	0.113		0.146	0.306	
	1.5	0.002	0.146		0.768	
	2	0.012	0.306	0.768		
**Re**	0.5		0.967	0.784	0.269	
	1	0.967		0.767	0.277	
	1.5	0.784	0.767		0.413	
	2	0.269	0.277	0.413		
**Os**	0.5		0.015	0.007	0.005	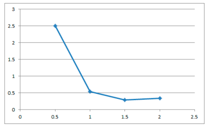
	1	0.015		0.495	0.122	
	1.5	0.007	0.495		0.527	
	2	0.005	0.122	0.527		

Table S3. Continued

	St. 2	0.5	1	2	3
**Ir**	0.5		0.087	0.092	0.058
	1	0.087		1.000	0.753
	2	0.092	1.000		0.846
	3	0.058	0.753	0.846	
**Pt**	0.5		0.264	0.158	0.411
	1	0.264		0.963	0.998
	2	0.158	0.963		0.920
	3	0.411	0.998	0.920	
**Au**	0.5		0.429	0.425	0.428
	1	0.429		0.003	0.978
	2	0.425	0.003		0.421
	3	0.428	0.978	0.421	
**Hg**	0.5		0.031	0.000	0.000
	1	0.031		0.044	0.013
	2	0.000	0.044		0.885
	3	0.000	0.013	0.885	
**Tl**	0.5		0.750	0.139	0.828
	1	1.414		0.437	1.000
	2	3.128	2.148		0.495
	3	1.209	0.052	2.010	
**Pb**	0.5		0.481	1.000	0.991
	1	2.041		0.687	0.708
	2	0.147	1.568		0.999
	3	0.422	1.517	0.224	
**Bi**	0.5		0.008	0.004	0.996
	1	0.008		0.411	0.763
	2	0.004	0.411		0.597
	3	0.996	0.763	0.597	

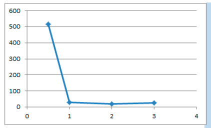
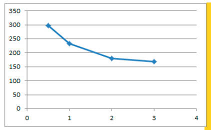
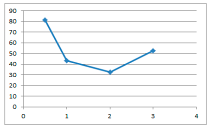

	St. 1	0.5	1	2	3	4
**Ir**	0.5		0.709	0.804	1.000	0.969
	1	0.709		0.992	0.754	0.694
	2	0.804	0.992		0.837	0.899
	3	1.000	0.754	0.837		0.977
	4	0.969	0.694	0.899	0.977	
**Pt**	0.5		0.009	0.001	0.025	0.414
	1	0.009		0.355	0.903	0.250
	2	0.001	0.355		0.336	0.066
	3	0.025	0.903	0.336		0.322
	4	0.414	0.250	0.066	0.322	
**Au**	0.5		0.214	0.096	0.223	0.269
	1	0.214		0.651	0.927	0.041
	2	0.096	0.651		0.747	0.018
	3	0.223	0.927	0.747		0.045
	4	0.269	0.041	0.018	0.045	
**Hg**	0.5		0.191	0.983	0.996	0.388
	1	0.191		0.937	0.912	0.014
	2	0.983	0.937		1.000	0.468
	3	0.996	0.912	1.000		0.622
	4	0.388	0.014	0.468	0.622	
**Tl**	0.5		1.000	0.993	0.945	0.787
	1	0.102		0.997	0.960	0.815
	2	0.589	0.490		0.997	0.929
	3	1.050	0.957	0.490		0.988
	4	1.606	1.529	1.131	0.691	
**Pb**	0.5		0.516	0.431	0.330	0.057
	1	0.516		0.155	0.118	0.017
	2	0.431	0.155		0.815	0.204
	3	0.330	0.118	0.815		0.304
	4	0.057	0.017	0.204	0.304	
**Bi**	0.5		0.005	0.132	0.734	0.702
	1	0.005		0.212	0.026	0.012
	2	0.132	0.212		0.297	0.126
	3	0.734	0.026	0.297		0.530
	4	0.702	0.012	0.126	0.530	

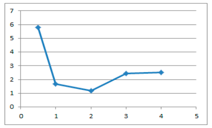
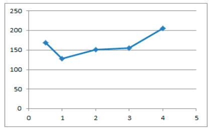
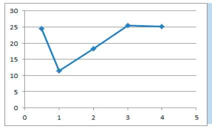

	St. 3	0.5	1	1.5	2
**Ir**	0.5		0.992	0.811	0.998
	1	0.992		0.867	0.984
	1.5	0.811	0.867		0.798
	2	0.998	0.984	0.798	
**Pt**	0.5		0.004	0.128	0.885
	1	0.004		0.193	0.019
	1.5	0.128	0.193		0.246
	2	0.885	0.019	0.246	
**Au**	0.5		0.063	1.000	0.722
	1	0.063		0.831	0.506
	1.5	1.000	0.831		0.793
	2	0.722	0.506	0.793	
**Hg**	0.5		0.309	0.488	0.758
	1	0.309		0.760	0.245
	1.5	0.488	0.760		0.376
	2	0.758	0.245	0.376	
**Tl**	0.5		0.034	0.208	0.650
	1	4.036		0.852	0.554
	1.5	2.825	1.139		0.934
	2	1.653	1.872	0.839	
**Pb**	0.5		0.122	0.840	0.873
	1	0.122		0.100	0.243
	1.5	0.840	0.100		0.745
	2	0.873	0.243	0.745	
**Bi**	0.5		0.029	0.108	0.873
	1	0.029		0.592	0.088
	1.5	0.108	0.592		0.223
	2	0.873	0.088	0.223	

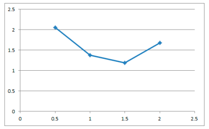
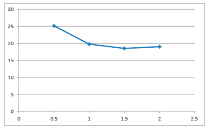

Table S3. Continued

	St. 2	0.5	1	2	3
**Th**	0.5		0.128	0.233	0.117
	1	0.128		0.991	0.815
	2	0.233	0.991		0.843
	3	0.117	0.815	0.843	
**U**	0.5		0.048	0.092	0.062
	1	0.048		0.988	0.959
	2	0.092	0.988		1.000
	3	0.062	0.959	1.000	

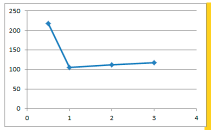

	St. 1	0.5	1	2	3	4
**Th**	0.5		0.971	0.654	0.639	0.005
	1	0.971		0.560	0.544	0.004
	2	0.654	0.560		1.000	0.463
	3	0.639	0.544	1.000		0.390
	4	0.005	0.004	0.463	0.390	
**U**	0.5		0.513	0.961	0.652	0.021
	1	0.513		0.565	0.465	0.023
	2	0.961	0.565		0.838	0.025
	3	0.652	0.465	0.838		0.760
	4	0.021	0.023	0.025	0.760	

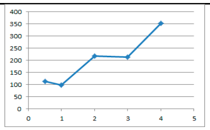

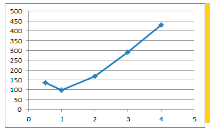

	St. 3	0.5	1	1.5	2
**Th**	0.5		0.096	0.002	0.506
	1	0.096		0.157	0.432
	1.5	0.002	0.157		0.038
	2	0.506	0.432	0.038	
**U**	0.5		0.015	0.078	0.009
	1	0.015		0.898	1.000
	1.5	0.078	0.898		0.868
	2	0.009	1.000	0.868	

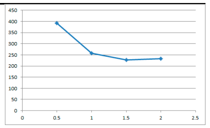

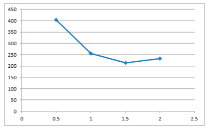

Table S4. Results of the three-way global and pairwise PERMANOVA tests on the variation of dry-to-wet weight ratio (*DW*/*WW*) in mussel soft tissues with age, location and sex

Source	df	SS	MS	Pseudo-F	P(perm)	Unique perms
Location	2	6.2189	3.1095	5.7164	0.005	999
Age	5	36.944	7.3888	13.584	0.001	999
Sex	1	0.40577	0.40577	0.74597	0.389	997
Location×Age**	5	3.4497	0.68994	1.2684	0.302	999
Location×Sex	2	1.2542	0.62709	1.1528	0.327	999
Age×Sex	5	0.48997	9.80E-02	0.18015	0.965	998
Location×Sex×Age**	5	2.5089	0.50179	0.92247	0.505	999
Res	94	51.132	0.54396			
Total	119	119				

PAIR-WISE TESTS

Term ' Location'				Term 'Age'			
Groups	t	P(perm)	Unique perms	Groups	t	P(perm)	Unique perms
St.1, St.2	1.816	0.061	998	0.5, 1	4.474	0.001	996
St.2, St.3	2.6534	0.01	999	0.5, 2	7.6353	0.001	997
St.1, St.3	1.9665	0.044	995	0.5, 3	6.1499	0.001	992
				0.5, 4	3.9177	0.003	999
				0.5, 1.5	2.6691	0.01	999
				1, 2	3.2928	0.003	997
				1, 3	2.5804	0.017	995
				1, 4	1.8512	0.076	998
				1, 1.5	1.0467	0.327	997
				2, 3	0.2779	0.798	996
				2, 4	1.4626	0.164	996
				2, 1.5	0.76741	0.503	997
				3, 4	0.1426	0.887	997
				3, 1.5	No test, df = 0		
				4, 1.5	No test, df = 0		

** Term has one or more empty cells

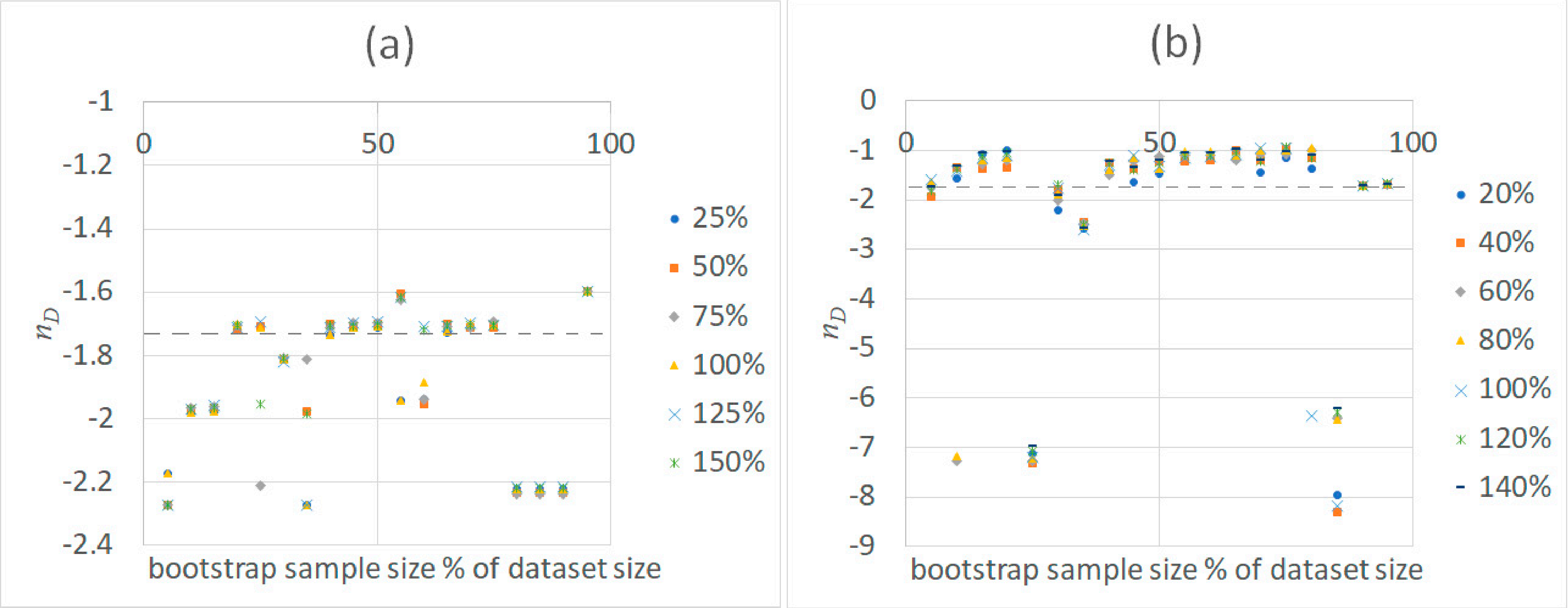

Figure S1. Finding the exponent of the function $F_1 = D^{n_D}$ significantly correlating with the contents of the largest number of elements: (a) Pearson's correlation and (b) Spearman's correlation. The bootstrap samples are random samples of fixed size (*x* axis), from which the most probable exponent values (with the largest occurrences) are extracted and accumulated in arrays of fixed size (percent of the dataset size, legend). The points are the most probable values in these arrays, and n_D is the median of all the values (dashed line).

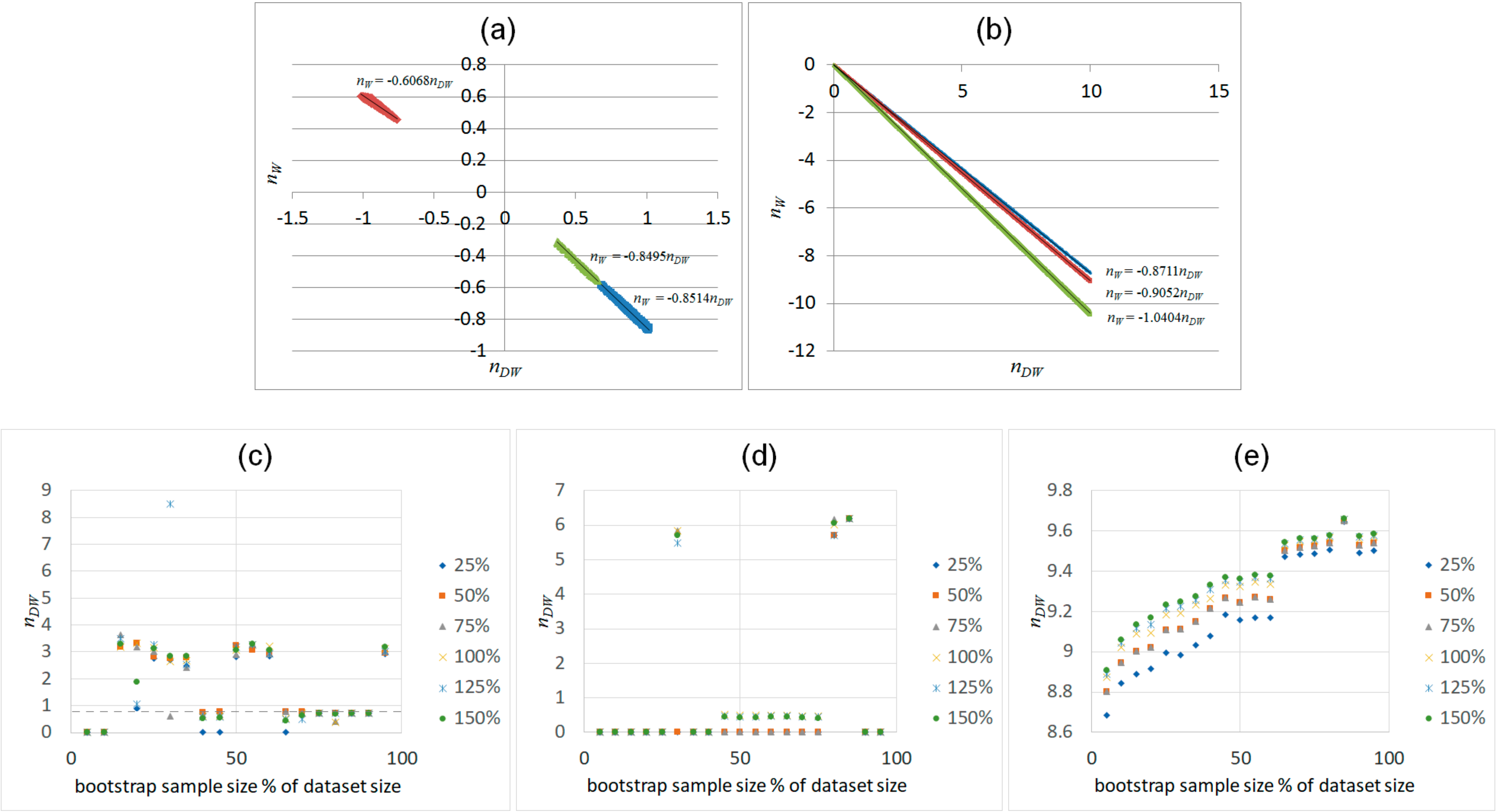

Figure S2. (a), (b) Relationships between n_{DW} and n_W of the function $F_2 = DW^{n_{DW}} W^{n_W}$ significantly correlating with the contents of the largest number of elements: (a) Pearson's correlation and (b) Spearman's correlation. (c) – (e) Bootstrapping procedure similar to that in Figure S1 for finding n_{DW}. Only the median $n_{DW} = 0.769$ (dashed line) in (c) satisfies the requirements and is used in the calculation of Spearman's n_W (blue line, $n_W = -0.8711 n_{DW}$).

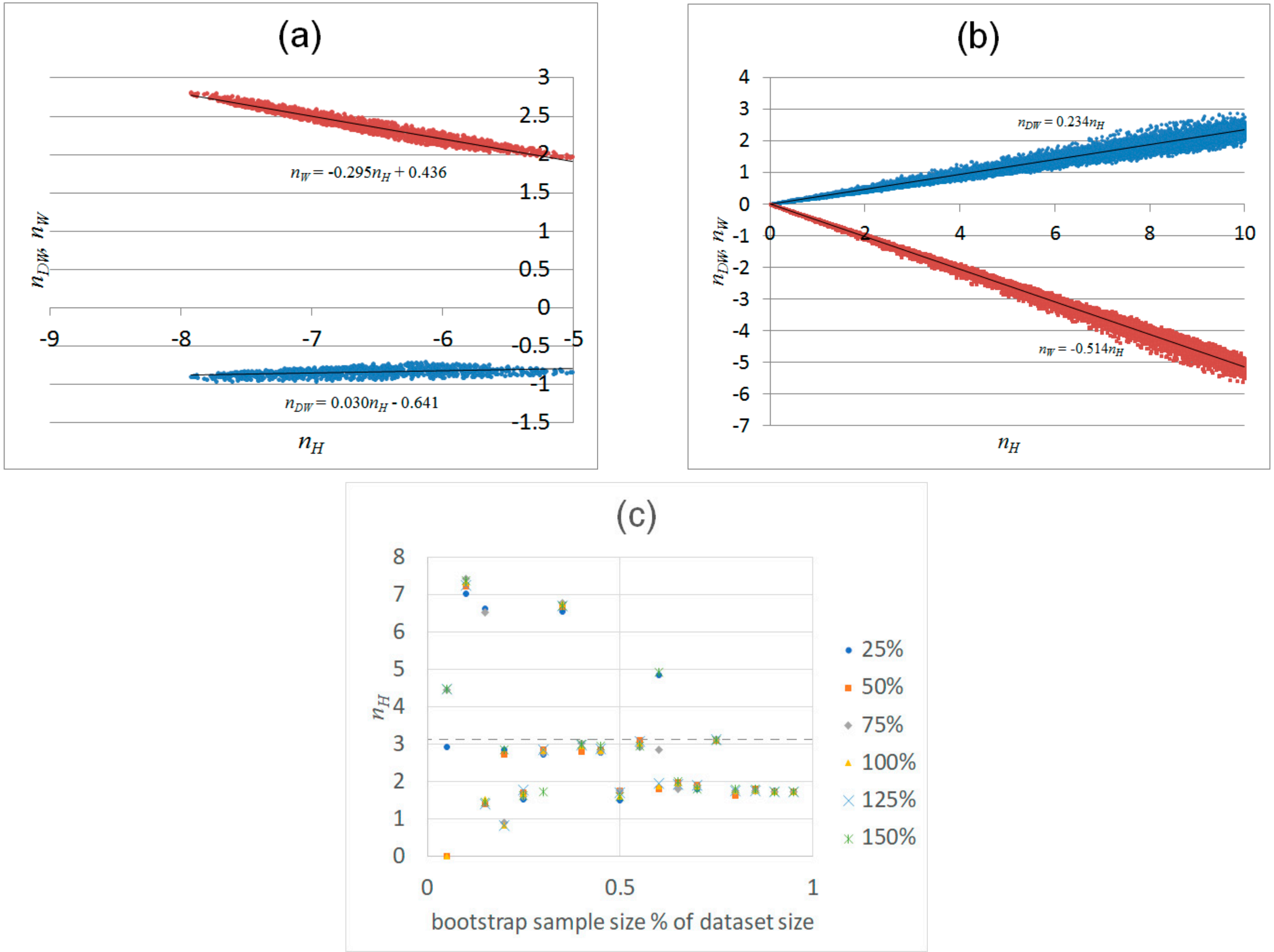

Figure S3. (a), (b) Relationships between n_{DW}, n_W and n_H of the function $F_3 = H^{n_H} DW^{n_{DW}} W^{n_W}$ significantly correlating with the contents of the largest number of elements: (a) Pearson's correlation and (b) Spearman's correlation. (c) Bootstrapping procedure similar to that in Figure S1 for finding n_H from a relationship in (b). The median $n_H = 3.126$ (dashed line) in (c) is used in the calculation of Spearman's n_{DW} (blue line, $n_{DW} = 0.234 n_H$) and n_W (brown line, $n_W = -0.514 n_H$).

Table S5. Pearson's and Spearman's correlation coefficients for different functions giving significant correlations ($P < 0.05$) with the largest number of element contents

	$1{-}DW/WW$		F_1		F_2		F_3	
	Pearson	Spearman	Pearson	Spearman	Pearson	Spearman	Pearson	Spearman
Li	0.30	0.24	0.41	0.39	−0.32	−0.29	0.34	−0.20
Be	0.24	0.21	0.33	0.28	−0.29	−0.34	0.25	−0.30
B	0.19		−0.18		−0.35	−0.37	0.29	−0.41
F	0.29	0.25			−0.47	−0.47	0.44	−0.54
Na	−0.19	−0.23	−0.31	−0.33	0.21	0.25	−0.26	0.27
Mg		−0.29	−0.42	−0.50	0.19	0.29	−0.25	0.28
Al	0.19		0.20	0.25	−0.18	−0.25	0.28	−0.22
Si	−0.26	−0.27			0.37	0.39	−0.37	0.46
P	−0.31	−0.29	−0.37	−0.40	0.47	0.43	−0.37	0.39
K	−0.35	−0.38	−0.20	−0.25	0.54	0.59	−0.48	0.62
Ca	0.26	0.27			−0.35	−0.50	0.38	−0.54
Sc	0.20				−0.20	−0.18	0.19	−0.19
Ti	0.23	0.30	0.24	0.33	−0.38	−0.48	0.38	−0.45
V	0.35	0.28	0.27	0.24	−0.45	−0.46	0.49	−0.46
Cr	0.29	0.20	0.27		−0.31	−0.33	0.28	−0.26
Mn			0.20	0.24			0.18	
Fe			0.27	0.23				
Co	0.29	0.27		0.22	−0.37	−0.42	0.35	−0.38
Ni	0.31	0.29	0.22		−0.36	−0.43	0.40	−0.38
Cu						−0.24		
Zn						−0.34		
Ga	0.26	0.24	0.23	0.26	−0.28	−0.43	0.37	−0.32
Ge	0.30	0.23			−0.40		0.38	−0.41
As	0.18		−0.31	−0.26				
Se					−0.27	−0.28	0.22	−0.27
Br		−0.23		−0.40		0.18	−0.18	0.18
Rb								0.19
Sr	0.26	0.21			−0.33	−0.43	0.35	−0.47
Y		0.23				−0.35		−0.40
Zr						−0.19		−0.21
Nb							0.22	−0.23
Mo	0.38	0.40	0.20		−0.33	−0.41	0.33	−0.41
Ru			0.22			0.23		0.26
Rh		0.33				−0.46		−0.43
Pd		0.27				−0.37		−0.39
Ag				0.25				0.18
Cd			0.25		0.19		−0.19	0.26
In		0.18	0.19	0.19		−0.27		−0.25
Sn		0.27				−0.37		−0.39
Sb		0.32		0.19		−0.37		−0.35
Te		0.29		0.22		−0.19	0.23	
I	0.21	0.31			−0.21	−0.33	0.19	−0.34
Cs	0.31	0.25	0.27	0.23	−0.38	−0.41	0.45	−0.41
Ba	0.22	0.26			−0.37	−0.47	0.36	−0.52

Table S5. Continued

	1-*DW/WW*		F_1		F_2		F_3	
	Pearson	Spearman	Pearson	Spearman	Pearson	Spearman	Pearson	Spearman
La			0.20	0.23	-0.19	-0.33		−0.28
Ce	0.27	0.24	0.28	0.35	-0.32	-0.35	0.36	−0.31
Pr	0.28	0.23	0.25	0.27	-0.35	-0.36	0.39	−0.34
Nd	0.33	0.32	0.19	0.24	-0.46	-0.54	0.48	−0.54
Sm	0.26	0.24	0.19	0.24	-0.36	-0.40	0.33	−0.37
Eu								
Gd	0.25	0.27			-0.42	-0.50	0.40	−0.50
Tb	0.34	0.32	0.22		-0.48	-0.53	0.50	−0.54
Dy	0.21	0.20	0.23	0.20	-0.29	-0.32	0.24	−0.26
Ho					0.18		−0.20	0.20
Er					-0.18	-0.22		−0.19
Tm					0.31	0.30	−0.31	0.35
Yb	0.24	0.20			-0.38	-0.41	0.35	−0.39
Lu	-0.20				0.35	0.29	−0.35	0.35
Hf		0.20		0.27		-0.21		−0.22
Ta				0.28				
W		0.31	0.20	0.29	-0.33	-0.49	0.34	−0.49
Re	0.28	0.39	0.18	0.19	-0.32	-0.50	0.26	−0.48
Os		0.36		0.21		-0.51	0.20	−0.56
Ir		0.21	0.19			-0.41	0.18	−0.44
Pt	0.23	0.29	0.19	0.23	-0.42	-0.62	0.36	−0.64
Au	0.29	0.29				-0.52	0.36	−0.56
Hg	0.34	0.31	0.22	0.20	-0.40	-0.44	0.40	−0.45
Tl								
Pb					-0.40	-0.40	0.34	−0.46
Bi	0.25	0.34		0.21	-0.37	-0.56	0.32	−0.59
Th						0.19		0.25
U			0.21	0.18				0.20

Multivariate analyses

Principal component analysis

Principal component analysis (PCA) seeks, using orthogonal transformation, the standardized linear combination of the original variables which has maximal variance [1,2]. To put it simplistically, this method looks for a few linear combinations of initial coordinates that can be used to present the data variance, with losing as little information as possible. This method reduces the total number of variables to the number of most significant – in terms of the resulting variance – ones.

The first step in this method is Z-standardization of the observations, which equalizes the weights of each variable X_i:

$$Z_{i,j} = \frac{X_{i,j} - \bar{X}_i}{\sigma_{X_i}} \tag{S1}$$

where $X_{i,j}$ is the *j*-th observation of *i*-th variable X_i, $\bar{X}_i$ is the mean value of this variable and σ_{X_i} is its standard deviation.

Next, a covariance matrix $\mathbf{A}$ is constructed on the basis of the standardized variables Z_i:

$$\mathbf{A} = \left[\operatorname{cov}\left(Z_{i=1..n}, Z_{k=1..n}\right)\right] = \begin{bmatrix} \operatorname{cov}(Z_1,Z_1) & \operatorname{cov}(Z_1,Z_2) & \cdots & \operatorname{cov}(Z_1,Z_n) \\ \operatorname{cov}(Z_2,Z_1) & \operatorname{cov}(Z_2,Z_2) & \cdots & \operatorname{cov}(Z_2,Z_n) \\ \vdots & \vdots & \ddots & \vdots \\ \operatorname{cov}(Z_n,Z_1) & \operatorname{cov}(Z_n,Z_2) & \cdots & \operatorname{cov}(Z_n,Z_n) \end{bmatrix}. \tag{S2}$$

The covariance of a variable with itself is its variance: $\operatorname{cov}(Z_i, Z_i) = \operatorname{var}(Z_i) = \sigma_{Z_i}^2$. The covariance matrix is symmetric since the covariance is commutative: $\operatorname{cov}(x, y) = \operatorname{cov}(y, x)$.

Then, eigenvectors and eigenvalues of the covariance matrix are calculated to determine the principal components. A non-zero vector $\mathbf{v}$ is eigenvector of the matrix $\mathbf{A}$ if the following equation holds:

$$\mathbf{Av} = \lambda \mathbf{v} \tag{S3}$$

where λ is some number called eigenvalue.

The eigenvalue problem is solved by equating the characteristic polynomial $p(\lambda)$ to zero:

$$p(\lambda) = \det(\mathbf{A} - \lambda \mathbf{I}) = 0 \tag{S4}$$

where $\mathbf{I}$ is the unit matrix of the same size as $\mathbf{A}$.

An equivalent notation for (S4) is

$$\begin{vmatrix} \operatorname{cov}(Z_1,Z_1)-\lambda & \operatorname{cov}(Z_1,Z_2) & \cdots & \operatorname{cov}(Z_1,Z_n) \\ \operatorname{cov}(Z_2,Z_1) & \operatorname{cov}(Z_2,Z_2)-\lambda & \cdots & \operatorname{cov}(Z_2,Z_n) \\ \vdots & \vdots & \ddots & \vdots \\ \operatorname{cov}(Z_n,Z_1) & \operatorname{cov}(Z_n,Z_2) & \cdots & \operatorname{cov}(Z_n,Z_n)-\lambda \end{vmatrix} = 0. \tag{S5}$$

Eigenvalues represent the variances for the principal components. Overall, n eigenvalues and eigenvectors exist for n variables. The matrix of column eigenvectors is composed of the solutions of the equations for each eigenvalue λ_i:

$$\left(\mathbf{A}-\lambda_i\mathbf{I}\right)\mathbf{v}_i=0\,. \tag{S6}$$

When arranged in the magnitude decrease order (alternatively, in the order corresponding to descending eigenvalues), these vectors form a system of principal components $\mathbf{V}$. The coordinates of these vectors are called loadings. The loadings are constrained to a sum of squares equal to 1.

To transfer data from the original coordinate system to the of, it is necessary to multiply the transposed matrix $\mathbf{V}$ by the transposed initial data matrix $\mathbf{X}$, in which observations are in lines:

$$\mathbf{Y}=\mathbf{V}^T\mathbf{X}^T \tag{S7}$$

where $\mathbf{Y}$ is the matrix of data in the principal components system, which are called scores.

The principal components do not only maximize the variance of the initial data, but also retain a property of being orthogonal to each other. In other words, the correlation between any pair of principal components is zero. The normalized principal component loadings for each variable and the principal component scores for each observation are often visualized in a single 2D or 3D plot (biplot) as vectors and points, respectively. The direction and length of the vectors show how much the variables contribute to the principal component in the plot.

Linear discriminant analysis

Linear discriminant analysis (LDA), also known as normal discriminant analysis, or discriminant function analysis, is another dimensionality reduction technique based on linear transformations. Unlike PCA focused on maximizing variance, it is aimed to find an orthogonal system of components that maximizes between-class separation at minimum within-class separation. To this end, the within-class and between-class scatter matrices are calculated.

The within-class scatter matrix $\mathbf{S}_w$ is defined as

$$\mathbf{S}_w=\sum_{k=1}^{C}\mathbf{S}_k \tag{S8}$$

where

$$\mathbf{S}_i=\sum_{\mathbf{x}\in D_i}^{n}\left(\mathbf{x}-\mathbf{m}_i\right)\left(\mathbf{x}-\mathbf{m}_i\right)^T \tag{S9}$$

is the scatter matrix for every class and

$$\mathbf{m}_i=\frac{1}{n_i}\sum_{\mathbf{x}\in D_i}^{n}\mathbf{x}_i \tag{S10}$$

is the mean vector for every class.

The between-class scatter matrix $\mathbf{S}_b$ is

$$\mathbf{S}_b = \sum_{i=1}^{C} N_i (\mathbf{m}_i - \mathbf{m})(\mathbf{m}_i - \mathbf{m})^T \tag{S11}$$

where $\mathbf{m}$ is the overall mean and N_i is the size of the respective class.

Next, a generalized eigenvalue problem is solved for the matrix $\mathbf{A} = \mathbf{S}_w^{-1}\mathbf{S}_b$ according to the steps described above in Eqs. (S3)-(S7).

The assumptions of LDA are normality and homoscedasticity of the data distribution. This allows constructing the within-group multivariate Gaussian probability density functions with class means $\mathbf{m}_i$ and a common covariance matrix $\mathbf{\Sigma}$:

$$f(\mathbf{x} \mid p_i) = \frac{1}{(2\pi)^{N/2} |\Sigma|^{1/2}} \exp\left[-\frac{1}{2} (\mathbf{x} - \mathbf{m}_i)^T |\Sigma|^{-1} (\mathbf{x} - \mathbf{m}_i) \right]$$

and determining the respective multidimensional ellipses of p_i probability of data in classes.

Canonical analysis of principal coordinates

In contrast to the above-mentioned ordination techniques dealing only with Euclidean or Mahalanobis distances, canonical analysis of the principal coordinates (CAP) can utilize any dissimilarity coefficients, e.g. the Bray-Curtis, Jaccard or Sørensen-Dice indices, as the measures of distance between vectors. Moreover, it is a constrained analysis in terms of the number of dimensions used.

Let $\mathbf{D} = (d_{ij})$ be the $N \times N$ matrix of distances from the data matrix $\mathbf{Y}$ with dimensions $N \times p$. Then, the matrix $\mathbf{A}$ can be defined [3] as $\mathbf{A} = (a_{ij}) = (-1/2 d_{ij})$. Next, Gower's centered matrix $\mathbf{G}$ can be calculated by centering the elements of $\mathbf{A}$:

$$\mathbf{G} = \left(\mathbf{I} - \left(\frac{1}{N} \right) \mathbf{1} \cdot \mathbf{1}^T \right) \mathbf{A} \left(\mathbf{I} - \left(\frac{1}{N} \right) \mathbf{1} \cdot \mathbf{1}^T \right) \tag{S12}$$

where $\mathbf{1}$ is the column vector of ones and $\mathbf{I}$ is an identity matrix.

For $\mathbf{G}$, the eigenvalue problem is solved, resulting in the matrix of orthonormal eigenvectors $\mathbf{Q}$. A subset of these eigenvectors (m) is selected to form a matrix ($\mathbf{Q}_m$) for the canonical analysis. To prevent overfitting of the model, m is kept small relative to N and the diagnostics for choosing appropriate dimensionality should be applied [4,5].

Next, the influence, or hat, matrix is calculated, which is idempotent: $\mathbf{H}^2 = \mathbf{HH} = \mathbf{H}$. In the present analysis, the hat matrix is

$$\mathbf{H} = \mathbf{X} \left[\mathbf{X}^T \mathbf{X} \right]^{-1} \mathbf{X}^T \tag{S13}$$

where $\mathbf{X}$ is the $N \times q$ matrix that contains either dummy variables as codes for groups (for the subsequent discriminant analysis) or quantitative variables (for canonical correlation analysis).

To relate the subset of m principal coordinate axes to $\mathbf{X}$, the matrix $\mathbf{Q}_m^T\mathbf{H}\mathbf{Q}_m$ is constructed, whose eigenvalue decomposition yields canonical eigenvalues δ^2 $\left(\delta_1^2, \delta_2^2, \ldots, \delta_s^2\right)$ and their associated eigenvectors. These canonical eigenvalues are also the squared canonical correlations. The number s of canonical axes is the minimum of $\left(m, q, (N-1)\right)$. The canonical test statistic is calculated as

$$tr\left(\mathbf{Q}_m^T\mathbf{H}\mathbf{Q}_m\right) \quad \text{(S14)}$$

When the canonical analysis is based on Euclidean distance and $m = p$, this is equivalent to the MANOVA test statistic known as Pillai's trace. The other test statistic is the first canonical eigenvalue δ_1^2, which is related to a statistic called Roy's greatest root criterion in traditional MANOVA. More precisely, Roy's criterion is $\delta_1^2 / \left(1 - \delta_1^2\right)$ if CAP is based on Euclidean distances and $m = p$.

By permuting original data within and between groups and finding the approximate distributions of either $tr\left(\mathbf{Q}_m^T\mathbf{H}\mathbf{Q}_m\right)$ or δ_1^2, one can calculate probabilities of validity of null hypothesis of no differences in the positions of the centroids among the groups in multivariate space.

References

1. Mardia, K.V.; Kent, J.T.; Bibby, J.M. *Multivariate Analysis*; Academic Press: London, 1979.
2. Rencher, A.C. *Methods of Multivariate Analysis*, 2nd ed.; Wiley: New York, 2002.
3. Hefner, J.T. Biological distance analysis, cranial morphoscopic traits, and ancestry assessment in forensic anthropology. In *Biological Distance Analysis: Forensic and Bioarchaeological Perspectives*, Pilloud, M.A., Hefner, J.T., Eds.; Elsevier: Amsterdam, 2016; pp. 301-315.
4. Anderson, M.J.; Willis, T.J. Canonical analysis of principal coordinates: a useful method of constrained ordination for ecology. *Ecology* **2003**, *84*, 511-525, doi:10.1890/0012-9658(2003)084[0511:CAOPCA]2.0.CO;2.
5. Anderson, M.J.; Gorley, R.N.; Clarke, K. *PERMANOVA+ for PRIMER: Guide to Software and Statistical Methods*; PRIMER-E: Plymouth, UK, 2008.
